# The Anti-Inflammatory Protein TNIP1 Is Intrinsically Disordered with Structural Flexibility Contributed by Its AHD1-UBAN Domain

**DOI:** 10.3390/biom10111531

**Published:** 2020-11-10

**Authors:** Rambon Shamilov, Olga Vinogradova, Brian J. Aneskievich

**Affiliations:** 1 Graduate Program in Pharmacology and Toxicology, Department of Pharmaceutical Sciences, University of Connecticut, Storrs, CT 06269, USA; rambon.shamilov@uconn.edu; 2 Department of Pharmaceutical Sciences, University of Connecticut, Storrs, CT 06269, USA; olga.vinogradova@uconn.edu

**Keywords:** intrinsically disordered regions, ABIN-1, linear motifs, NMR, natively unfolded protein

## Abstract

TNFAIP3 interacting protein 1 (TNIP1) interacts with numerous non-related cellular, viral, and bacterial proteins. TNIP1 is also linked with multiple chronic inflammatory disorders on the gene and protein levels, through numerous single-nucleotide polymorphisms and reduced protein amounts. Despite the importance of TNIP1 function, there is limited investigation as to how its conformation may impact its apparent multiple roles. Hub proteins like TNIP1 are often intrinsically disordered proteins. Our initial in silico assessments suggested TNIP1 is natively unstructured, featuring numerous potentials intrinsically disordered regions, including the ABIN homology domain 1-ubiquitin binding domain in ABIN proteins and NEMO (AHD1-UBAN) domain associated with its anti-inflammatory function. Using multiple biophysical approaches, we demonstrate the structural flexibility of full-length TNIP1 and the AHD1-UBAN domain. We present evidence the AHD1-UBAN domain exists primarily as a pre-molten globule with limited secondary structure in solution. Data presented here suggest the previously described coiled-coil conformation of the crystallized UBAN-only region may represent just one of possibly multiple states for the AHD1-UBAN domain in solution. These data also characterize the AHD1-UBAN domain in solution as mostly monomeric with potential to undergo oligomerization under specific environmental conditions (e.g., binding partner availability, pH-dependence). This proposed intrinsic disorder across TNIP1 and within the AHD1-UBAN region is likely to impact TNIP1 function and interaction with its multiple partners.

## 1. Introduction

TNFAIP3 interacting protein 1 (TNIP1) (also known as ABIN-1, NAF or VAN) is a multi-domain, non-enzymatic regulator of cytoplasmic and nuclear cellular signaling. Initially identified as an inhibitor of NF-kB in conjunction with its binding partner A20 [1,2], numerous not-necessarily related interaction partners of TNIP1 have been reported including HIV proteins Nef and Matrix [3,4], ERK2 [5], ligand-bound PPARs and RARs [6,7], NEMO [8], HDAC1 [9], NLRP10 [10] and the Shigella IpaH9.8 protein [11]. The promiscuous nature of TNIP1 suggests its existence as a hub protein (defined here as having ≥10 partners [12]. A common feature shared by hub proteins is a greater degree of intrinsic structural disorder (i.e., natively unfolded conformation, structural flexibility) [13]. The vast potential for clinical importance of intrinsically disordered proteins (IDPs) has been established by recognizing their integral role in numerous signaling and other control pathways (e.g., p53, p21, BRCA1) [14,15]. Potential TNIP1 roles in normal cell physiology and several inflammatory and autoimmune pathologies have been identified through numerous genome-wide association studies, as reviewed in [16,17]. Decreased levels of TNIP1 protein have been reported in human autoimmune and inflammatory diseases such as systemic sclerosis and psoriasis [18,19]. Perhaps modeling this, TNIP1 protein degradation is enhanced in response to inflammatory signaling from interleukin-17 stimulation in vitro [20]. While many endpoints of TNIP1 function have been reported, there is to-date limited investigation of TNIP1 protein characteristics that may contribute to its control over intracellular signaling [21,22]. Further investigation of TNIP1 protein, specifically as a candidate IDP, might help explain its diverse partner interactions or degradation sensitivity, and could yield potential translational human health benefits in diverse pathologies.

An extended repertoire of protein–protein interactions based on intrinsic disorder does not require conformational flexibility to extend throughout the entirety of one of the partner proteins. Intrinsically disordered protein regions (IDPR) are localized sections lacking fixed secondary or tertiary structure. These IDPRs and other subregions of a wholly disordered protein commonly facilitate high-specificity, low affinity interactions [23,24]. Among the many regions for partner interaction along the TNIP1 protein is the ubiquitin binding domain (UBD) (later named UBD in ABIN proteins and NEMO, UBAN [25]), which facilitates TNIP1 interaction with polyubiquitin chains [26]. TNIP1–partner interactions also include the involvement of additional amino acids in the ABIN homology domain 1 (AHD1) upstream of the UBAN, which function in tandem with it to promote binding of the deubiquitinase/ubiquitin ligase protein A20 and polyubiquitin. With the intervening amino acids linking the AHD1 and UBAN domains, they span approximately from AA417 through 509 in the human protein sequence. In part via TNIP1–partner interactions mediated within this region, TNIP1 maintains proportional inflammatory responses through dampening of intracellular signaling downstream of activated membrane receptors e.g., tumor necrosis factor receptor and toll-like receptor ([16] for review). This region is also within an extended TNIP1 fragment of ~230 amino acids which when expressed via transient transfection reduced TNF-stimulated reporter expression [1]. Further truncations of the fragment, retaining the UBAN homology region but excluding its upstream AHD1 and the domain linking that to the UBAN region, were used to establish in vitro interaction with ubiquitin [25]. An aspartic acid to asparagine point mutation in the TNIP1 UBAN domain results in loss of polyubiquitin binding. When expressed in mice as a knockin gene replacing the wild-type sequence, the ubiquitin-binding defect promotes spontaneous lupus-like autoimmune disease [26]. Notably however, TNIP1 can function in signal repression even in the absence of A20 [27], suggesting innate properties or other as of yet unrecognized interaction partners.

IDPRs are often defined by molecular recognition features (MoRFs). These regions can be intrinsically disordered under basal conditions and undergo disorder-to-order transition taking on alpha helical, beta strand, or coiled conformations through interaction with a binding partner [24]. Additionally notable, is that retention of a partially unstructured conformation upon binding with self or partner proteins have also been reported [28,29,30,31,32]. As a candidate hub IDP, such properties can be expected along TNIP1 and involved in interactions with its partner proteins. Modeling these for TNIP1 is limited as a crystal structure of human full-length sequence or monomeric TNIP1 UBAN has yet to be reported. However, crystal structure studies of the UBAN domain of mouse TNIP1 [21] and a UBAN fragment with its upstream AHD1 from the TNIP1-related human TNIP2 (i.e., ABIN2) [22] indicate these regions exist as a MoRF which can form a self-associated coiled-coil dimer, bound and unbound in the case of mouse TNIP1 UBAN, by polyubiquitin. The small cohort of TNIP1-related proteins, i.e., TNIP2 (ABIN-2) and TNIP3 (ABIN-3), are more functionally related in their repression of NF-kB-mediated signaling than in their sharing protein sequence. They show minimal consensus across their protein length (TNIP2, 429AA; TNIP3, 325AA) with it mostly localized to very limited matches in aligned subdomains, such as AHD1 and UBAN [22,33]. If the AHD1 and UBAN domains are IDPRs, these crystal structures would be informative for a static image representing one phase in a possible conformational continuum in this protein region. This protein region may exist within a cohort of previously reported substantially disordered proteins that gain sufficient order in a bound form that allows for crystallization [34]. Determination of intrinsic disorder traits along the full-length TNIP1 or especially within the UBAN domain could guide further investigation on TNIP1 partner interactions and their consequences to repression of intracellular signaling.

The characteristic compositional and biophysical aspects of intrinsic disorder, including increased charge/polarity, reduced hydrophobic core formation, and increased dynamic protein backbone promotes unique challenges in expression and investigation of these proteins [35,36]. Databases of intrinsically disordered proteins such as DisProt [37], have allowed for extensive development of algorithms for prediction of disorder [38]. With some of its functional and cellular characteristics reminiscent of IDP, e.g., multiple partners and increased degradation under cell stress conditions, we undertook comprehensive computational and biophysical testing of TNIP1 as an IDP. In silico analysis of TNIP1 and the AHD1-UBAN domain by such algorithms reveals predicted intrinsic disorder, consistent with increased polar/charged amino acid residues. We found that the in silico predictions and bioinformatics approach reliably foreshadowed our in vitro results. Using methods effective at characterizing protein shape (e.g., dynamic light scattering (DLS), analytical ultracentrifugation (AUC), analytical gel filtration), secondary and tertiary structure characteristics (e.g., limited proteolysis, circular dichroism (CD)) and protein dynamics (e.g., NMR) we present data indicating that both the full-length TNIP1 and TNIP1 AHD1-UBAN regions are extended, non-structured proteins. As a potential IDPR of interest within TNIP1, we found the AHD1-UBAN region will undergo conformational shifts with appropriate stimulus (i.e., increased concentration, pH shift and addition of binding partner). This distinct investigative approach suggests anti-inflammatory, TNIP1-mediated signal repression is occurring in the context of, and may be dependent on, an unstructured flexible protein conformation. Additionally, the results emphasize the possible contribution of IDP in general to regulation of inflammatory signaling in addition to the other already well-established pathologies where IDP can be key proteins [14,39].

## 2. Materials and Methods

### 2.1. In Silico Analysis

Sequences for full-length TNIP1 were collected from UniProt release 2020_05 [40] (www.uniprot.org) (human sequence-Q15025, for a complete list of species used see supplementary materials). For review of protein disorder prediction algorithms, see [41,42,43]. For determining disorder scores of human full-length TNIP1 and TNIP1 AHD1-UBAN domains (AA417-509), web-based algorithms VL-XT, VL3, VSL2, IUPred2, MFDp2 and PONDR-FIT were used (available at http://www.pondr.com/ for VL-XT, VL3 and VSL2; https://iupred2a.elte.hu for IUPred2; http://biomine.cs.vcu.edu/servers/MFDp2/ for MFDp2; http://original.disprot.org/pondr-fit.php for PONDR-FIT). For the above algorithms, residues with scores above 0.5 are considered disordered and below 0.5 are considered ordered. Prediction of post-translation modifications was performed using D2P2 (available at http://d2p2.pro/). Prediction of coiled-coil domains for full-length TNIP1 was performed using DeepCoil [44] (available at https://toolkit.tuebingen.mpg.de/tools/deepcoil). Amino acid enrichment or depletion presented as fractional difference in composition was determined with Composition Profiler (available at http://www.cprofiler.org/index.html) using either the full-length TNIP1 or TNIP1 AHD1-UBAN region sequence compared against SwissProt database. To establish a trend of amino acid compositional bias in IDPs, a similar comparison of DisProt versus SwissProt databases was done. Cumulative distribution function (CDF) was performed in a similar manner as the above predictors (available at http://www.pondr.com). CDF plot represents the fraction of residues within an amino acid region that are scored at a given value. Largely disordered proteins will have low numbers of residues with low scores and vice versa for ordered proteins. Within a CDF plot, disordered proteins will appear mainly below the boundary line (black-dotted) and ordered proteins above. Proteins which cross the boundary line are considered a mixture of disordered and ordered. Boundary points for the CDF plot were obtained from the PONDR server. Charge–hydropathy (CH) analysis presented as absolute mean net charge versus mean scaled hydropathy plotted using provided ordered and disordered data sets as well as threshold values as established by Uversky et. al. [45] available at http://www.pondr.com/. Species homology analysis of full-length TNIP1 using default parameters of the Clustal Omega sequence alignment [46] program (available at https://www.ebi.ac.uk/Tools/msa/clustalo/) for three or more sequences analyzed. Gaps introduced by Clustal Omega were then incorporated in the PONDR-FIT raw data scores for each species to allow plotting of all species’ PONDR-FIT data to reflect sequence alignments. Percent identity matrix scores were exported as part of results generated with use of Clustal Omega algorithm.

### 2.2. Recombinant Protein Expression and Purification

pET28a-TNIP1 (full length and TNIP1 AHD1-UBAN construct (TNIP1^417-509^)) featuring a C-terminus His6 tag were transformed into BL-21(DE3) Rosetta 2 host strain *E. coli* (Millipore Sigma, Burlington, MA, USA). Expression was performed by diluting overnight inoculated LB grown to saturation at a 1/40 (*v/v*) dilution into LB. Diluted cultures were agitated at 37 °C, 250 RPM until an OD600 of ~0.2–0.3 was reached after which cultures were induced with 0.1 mM isopropyl-b-D-thiogalactopyranoside (IPTG) at 30 °C for 4 h. Cultures were then pelleted at 4000× *g* for 20 min before storage at −20 °C. Purification of pET28a-TNIP1 (full length and TNIP1^417-509^) was performed by resuspending bacterial pellets 1/20 (*w*/*v*) in 20 mM sodium phosphate buffer (pH 8.0), 500 mM NaCl, and 20 mM imidazole and lysing the pellets by placing heating in a boiling water bath for 20 min followed by immediate cooling on ice for 10 min. This lysis scheme, as established for other IDPs which remain soluble with heat treatment [47,48,49,50,51], was used to overcome poor yields from non-heat lysis methods. Lysates were clarified at 28,000× *g* for 35 min followed by filtering through a 0.45 micron filter before injection onto a 5 mL HisTrap HP column (GE Healthcare Life Sciences, Marlborough, MA, USA) at 1 mL/min. The column was then washed with 5 column volumes 20 mM sodium phosphate buffer (pH 8.0), 500 mM NaCl, and 40 mM imidazole and eluted in 20 mM sodium phosphate buffer (pH 8.0), 500 mM NaCl, and 300 mM imidazole. Samples were then either immediately snap frozen for storage at −80 °C or used for further purification by concentration via Amicon Ultra Centrifugal Filter Units (Millipore Sigma) and injection onto a HiLoad Superdex 75 16/60 HP column (GE Healthcare Life Sciences) pre-conditioned overnight with 20 mM sodium phosphate buffer (pH 8.0) and 200 mM NaCl. Purity of protein assessed at each stage of purification via SDS-PAGE gel analysis using ImageJ [52] and protein quantification performed using a Pierce 660 nm Protein Assay (ThermoFisher Scientific, Waltham, MA, USA). Molecular weight determination was performed using a Kodak IS400CF CCD imager (Kodak, Rochester, NY, USA) and Carestream Molecular Imaging Software. Western blot analysis was performed by transfer of electrophoretically resolved protein samples onto a Whatman Protran nitrocellulose membrane (GE Healthcare Life Sciences, Marlborough, MA, USA). Blots were then incubated with 5% nonfat dry milk in tris-buffered saline with 0.05% Tween-20 (TBST) for 1 h at room temperature followed by incubation with anti-His6 antibody (1:1000 dilution, Cell Signaling Technology, catalog number: 2366). Blots were washed with TBST before a 2-h incubation with HRP-conjugated anti-mouse secondary antibody (1:5000 dilution, Abcam, catalog number: ab97046). Proteins were imaged using chemiluminescence substrates (ThermoFisher Scientific, Waltham, MA, USA) and a Kodak IS440CF CCD imager (Kodak, Rochester, NY, USA).

### 2.3. Analytical Gel Filtration

Purified TNIP1^417-509^ construct at 1 mg/mL was injected onto a HiLoad Superdex 75 16/60 HP column preconditioned overnight with 20 mM sodium phosphate buffer (pH 8.0), and 200 mM NaCl. The column was run at 0.8 mL/minute and 2 mL fractions were collected over the course of the gel filtration run. TNIP1 elution volume was monitored by absorbance at 220 nm and 230 nm. The elution volume was plotted on a standard curve (volume of elution versus molecular weight (kDa)) of protein standards injected separately under the same conditions. The protein standards used were bovine serum albumin (BSA), ovalbumin, myoglobin, chymotrypsinogen, and cytochrome C. For comparison of hydrodynamic radii versus molecular mass, random coil and pre-molten globule-like protein standards were taken from [53] and globular proteins were taken from [54].

### 2.4. Light Scattering

The hydrodynamic radius and molecular weight of purified TNIP1^417-509^ construct was measured simultaneously using a Zetasizer Nano ZSP (Malvern Panalytical, Malvern, UK) at 1.5 and 0.75 mg/mL in 20 mM sodium phosphate buffer (pH 8.0), 200 mM NaCl and 10% glycerol (included to reduce aggregates). Molecular weight measurements were made using static light scattering (SLS) comparing TNIP1^417-509^ versus a toluene scattering standard. Four dilutions of TNIP1^417-509^ were used with samples prepared in a quartz cuvette (Malvern Panalytical) for molecular weight determination. Protein samples were prepared with centrifugation at 10,000× *g* for 10 min followed by filtering through a 0.2 micron filter. DLS data are presented as total percentage by volume, converted from total percentage by intensity. Heterogeneity of the samples was determined by a measured polydispersity index (PDI) obtained via DLS experiments.

### 2.5. Analytical Ultracentrifugation (AUC)

Purified TNIP1^417-509^ protein samples were prepared in 20 mM sodium phosphate buffer and 200 mM NaCl at either pH 5.8 or 8.0 by extensive buffer exchange using Amicon Ultra Centrifugal Filter Units reaching a minimal dilution factor of 1/1000. Sedimentation velocity analysis was conducted at 20 °C and 50,000 RPM using absorbance optics with a Beckman-Coulter XL-I (Beckman Coulter, Brea, CA, USA) analytical ultracentrifuge. Double sector cells equipped with quartz windows were used. The rotor was equilibrated under vacuum at 20 °C and after a period of ~30 min at 20 °C the rotor was accelerated to 50,000 RPM. Absorbance scans at 230 nm were acquired at 6 min intervals for ~20 h. Protein physical constants, buffer density and viscosity were determined using Sednterp. Data analysis was performed in Sedfit using a continuous sedimentation coefficient distribution (c(s)). The c(s) analyses were done at a resolution of 0.05 S, using maximum entropy regularization with a 95% confidence limit.

### 2.6. Limited Proteolysis

Protein was subjected to proteolysis under limiting protease conditions (1:1000, 1:2500 *w*/*w* protease: TNIP1^417-509^ and 1:250 *w*/*w* protease: full-length TNIP1 at 25 °C or 37 °C with collection of samples at 1, 5, 10, 15, and 30 min. Commercially available BSA (Thermo Fisher Scientific, Waltham, MA, USA) and alpha-casein (Millipore Sigma) were used in control experiments. Trypsin and chymotrypsin diluted from freshly made stocks were used for limited proteolysis experiments. Proteolysis was performed in 20 mM sodium phosphate buffer (pH 8.0), 200 mM NaCl, 2 mM CaCl for TNIP1^417-509^ and 20 mM sodium phosphate buffer (pH 8.0), 500 mM NaCl, 200 mM imidazole for studies using full-length TNIP1. Quenching of protease digestion was by addition of 5× Laemmli protein sample buffer and immediate heating at 95 °C for 5 min prior to resolving digest fragments by SDS-PAGE.

### 2.7. Circular Dichroism

CD spectra were collected on a Chirascan V100 spectropolarimeter (Applied photophysics, Surrey, UK) scanning from 190–250 nm with a 1 nm step and 2 nm bandwidth. 400 µL of TNIP1^417-509^ protein at a final 10 µM (0.13 mg/mL) concentration in 50 mM sodium phosphate buffer (pH 8.0) scanned using a quartz cuvette with a 1 mm pathlength (Starna Cells, Atascadero, CA, USA). All spectra were blanked against spectra collected from buffer-only samples. Scans done at increasing temperatures were performed using a Precision Peltier (Quantum Northwest, Liberty Lake, WA, USA) temperature controller. Secondary structure characterization studies were performed using protein samples under the same conditions as above with the addition of 10, 20, 30 or 40% (*v*/*v*) 2,2,2-trifluoroethanol (TFE) for 1 h prior to scanning. Post CD analysis done using the DichroWeb server using the Contin-LL (Provencher and Glockner Method) using database 7 from [55] and references therein. Standards for comparison of AHD1-UBAN versus random coil and pre-molten globule proteins were taken from [45].

### 2.8. Protein Expression for Nuclear Magnetic Resonance (NMR) Studies

^15^N labeled protein was expressed in minimal media consisting of 95 mM KH2PO4, 57 mM K2HPO4, 63 mM Na2HPO4, 13 mM K2SO4, 10 mM MgCl2, 0.2 mM CaCl2, 0.1 mM thiamine HCl, 13 mM EDTA, 0.4% glucose, 1× MEM vitamin solution (Thermo Fisher Scientific), 1× Trace Metal Mixture (Thermo Fisher Scientific). ^15^N labeling was achieved with inclusion of 20 mM (1 g/L) ^15^NH4Cl in expression media. Monoubiquitin (pET15b-ubiquitin) was expressed in LB media after induction with 0.1 mM IPTG at 37 °C of cultures, post-recovery after 1/40 (*v*/*v*) inoculation from saturated overnight culture, reaching an OD600 of 0.4–0.5. Induction lasted for 6 h before the culture was spun down at 4000× *g* for 20 min before storage at −20 °C. pET15b-ubiquitin pellets were lysed using sonication in 20 mM sodium phosphate buffer (pH 8.0), 500 mM NaCl, and 20 mM imidazole with complete, Mini, EDTA-free protease inhibitor cocktail (Millipore Sigma), 20 mg/mL lysozyme, and 5 mg/mL DNase 1. Sonicated lysates were clarified and then purified by affinity chromatography and gel filtration as described above.

### 2.9. NMR Spectroscopy

All NMR experiments were performed on a Varian Inova 600 MHz spectrometer equipped with a cryogenic probe (Agilent, Palo Alto, CA, USA). ^1^H–^15^N heteronuclear single quantum coherence (HSQC) experiments were performed to collect TNIP1^417-509^ (0.3 M) alone at pH 6.6, 5.8 and TNIP1^417-509^ plus monoubiquitin (1:2.2 molar ratio) spectra in 20 mM sodium phosphate buffer, 50 mM NaCl with 10% D2O at 25 °C. Data analysis performed using programs made available in NMRBox [56].

## 3. Results

### 3.1. In Silico Analysis of Full-Length Human TNIP1 and Species Homologues

**Figure 1:**
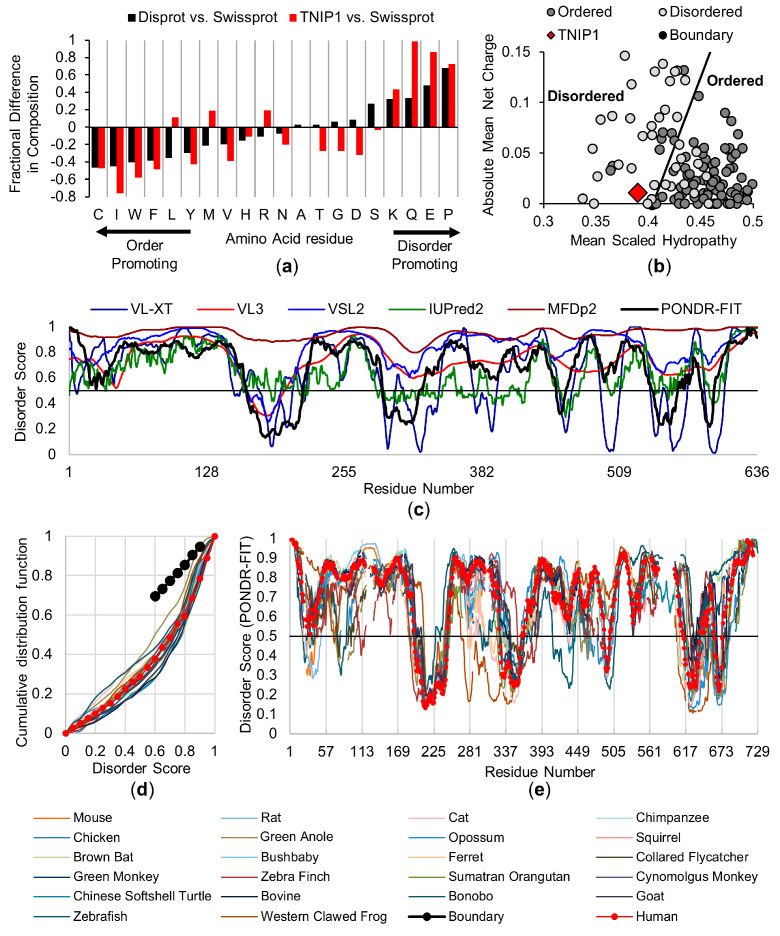
In silico analysis of TNIP1 highlights intrinsic disorder characteristics. (**a**) Amino acid compositional bias of full-length TNFAIP3 interacting protein 1 (TNIP1) (shown in red) generated comparing TNIP1 versus database of globular proteins (Swissprot) overlaid as comparison against database of disordered proteins (DisProt) versus SwissProt (shown in black). (**b**) Charge–hydropathy plot of full-length TNIP1 (red diamond) with ordered (dark grey circles) and disordered (light grey circles) plotted with boundary representing likely delineation of trends in charge and hydrophobicity between plotted proteins. (**c**) Full-length TNIP1 (636AA) disorder predictions as generated by independent algorithms where disorder score above 0.5 threshold indicates prediction of disorder. (**d**,**e**) Colored lines represent 23 species that were used for comparison to human (dotted red line) (**d**) Cumulative distribution function (CDF) with 23 species inputs plotted along with boundary (dotted black line) separating potential disordered (found mostly below line) from ordered proteins (found above line). (**e**) PONDR-FIT-fit analysis of 23 species (human—dotted red line) after alignment using Clustal Omega. Gaps introduced after amino acid sequence alignment maintained in graphed data.

IDPs and IDPRs are conformationally distinct from most proteins due to a general lack of fixed three-dimensional structure, generated by low abundance of hydrophobic amino acids which typically promote tight, hydrophobic cores [57,58]. Full-length TNIP1 (636AA) is characterized by a skewed distribution of amino acids with an increase in disorder promoting (Lys, Glu, Gln, Pro) and decrease in order-promoting (Cys, Ile, Trp, Phe, Leu, Tyr) residues (Figure 1a). Disordered proteins commonly having high net charge and reduced hydrophobicity [49,59]. This allows for segregation of known disordered and ordered protein standards and predicts a protein’s intrinsic disorder based on where it lies on the spectrum (Figure 1b) of a charge–hydropathy plot. Full-length TNIP1 is grouped with the disordered proteins left of the threshold due to compositional bias favoring relatively increased numbers of hydrophilic residues and decreased hydrophobic content. Ongoing biophysical identification of disordered proteins and protein regions is improving an ever-growing number of effective disorder predictor algorithms [41,42,43], particularly those (i.e., meta-predictors) outputting collective assessment from multiple, individual programs [38]). Potential disorder for the full-length human TNIP1 protein was assessed using four standalone algorithms (VL-XT, VL3, VSL2 and IUPred2) and two meta-predictors, (MFDp2 and PONDR-FIT), in which a score above an 0.5 threshold for any amino acid residue is indicative of intrinsic disorder (Figure 1c). Looking at the residue scores generated using PONDR-FIT (reported to be ~11% more accurate than individual predictors [60]), there are reported potential regions of order (residue scores below 0.5) across amino acids regions of ~165–216, 291–327, 451–460, 539–596. This leaves a greater portion of the protein above the 0.5 threshold (average residue score being 0.671 across the entire TNIP1 protein). This trend is consistent across the standalone algorithms, where the average residue scores track closely with the meta-predictor PONDR-FIT (VL-XT-0.64, VL3-0.73, VSL2-0.83, IUPred2-0.64, MFDp2-0.95). Thus, there is very good agreement among the different platforms of a high degree of disorder across a high percentage of the full-length TNIP1 protein, with several extended lengths above the 0.5 threshold, i.e., suggestive of a mostly disordered protein with numerous IDPR (Figure 1c).

Intrinsic disorder contributing to protein functionality could be expected to be present across species protein homologues [61]. To test whether this is true for TNIP1 across species homologues, we generated a multiple sequence alignment of 23 full-length proteins (Supplemental Table S1) from various species using Clustal Omega [46] (Supplemental Figure S1). To illustrate the similarities in predicted disorder among these different species, we used a cumulative disorder fraction (CDF) algorithm (Figure 1d), which plots determined disorder scores versus the frequency of occurrence. Across all species assessed, we see the plotted values lying below the threshold (Figure 1d—dotted black line) which would indicate all these sequences belong to disordered proteins. Alignments generated through Clustal Omega were used to inform gaps within the overlaid PONDR-FIT assessment of the 23 species (Figure 1e). Across the assessed species, there is a similarity in TNIP1 protein disorder, with PONDR-FIT scores similarly trending across the different species when compared to the human sequence (Figure 1e—red-dotted line), consistent with the CDF output. The regions of disorder and predicted order are mostly conserved with traces overlapping and average PONDR-FIT scores ranging from 0.62–0.71 across the species. This is reflected in the comparison of the human TNIP1 PONDR-FIT score versus the average of all species used in the amino acid sequence alignment (Supplemental Figure S2A). Unsurprisingly, the five most similar to human in amino acid sequence identity (Supplemental Table S2A) mirrored the human PONDR-FIT disorder score (Supplemental Figure S2B). The species with less than 60% matched identity still showed similar predicted disorder and ordered regions of full-length TNIP1 (Supplemental Figure S2C). This, as well as the PONDR-FIT score similarities, is consistent with reports of predicted structural flexibility being conserved looking at families of proteins and protein domains [62] as well as protein homologs across diverse species with low conserved amino acid identity [63].

### 3.2. Full-Length TNIP1 Expression and Limited Proteolysis

**Figure 2:**
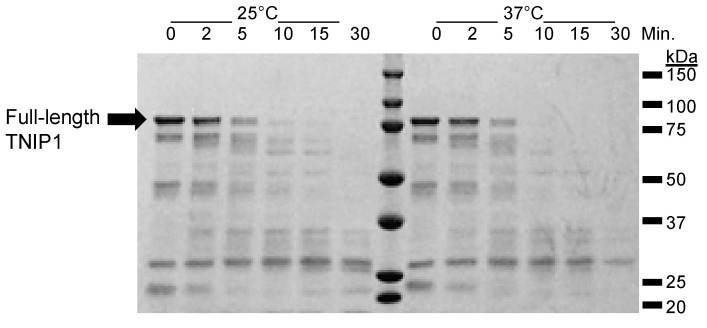
Limited proteolysis of full-length TNIP1. Limited proteolysis performed with full-length TNIP1 with 1:250 trypsin to protein ratio (*w*/*w*) at 25 °C and 37 °C. Samples collected up to 30 min at times indicated at top of lane. Reactions were quenched by adding 5× Laemmli buffer and heating at 95 °C for 5 min prior to loading 6 μg for analysis by SDS-PAGE.

Structural flexibility and lack of tight, packed hydrophobic regions in IDPs introduces unique challenges to expressing and purifying recombinant IDPs. Namely, with increased peptide surface exposure to the environment, IDPs are increasingly more sensitive to protease digestion by bacterial host enzymes [35,36,64,65]. Recombinant expression of full-length TNIP1 (pET28a-TNIP1 with C-terminus His6 tag) resulted in poor yields of purified protein under typical conditions (lysis by sonication with protease inhibitors present, data not shown), most likely due to degradation by proteases active in the bacteria becoming released with lysis and being retained through purification. However, taking advantage of an often IDP-associated property of thermal stability at high temperatures in solution [47,48,49,50,51], we are able to obtain an enriched protein TNIP1 band at ~81 kDa (by gel scanning analysis) (Supplemental Figure S3A). Notably, this apparent SDS-PAGE gel molecular weight from migration standards is higher than the predicted 73.92 kDa (71.82 kDa TNIP1 plus 2.1 kDa linker/HIS6 tag). This is not unexpected as this increased shift in molecular weight, often attributed to be due to poor interactions with SDS, is commonly seen with proteins featuring compositional bias typical of IDPs (increased net charge and reduced hydrophobicity) [66]. Affinity purification of full-length TNIP1 resulted in retrieval of the presumed full-length protein, as well as a pattern of smaller protein products (Supplemental Figure S3B). The larger band was confirmed as full-length TNIP1 by LC-MS/MS, with the smaller band identified as His6 tagged proteins by Western blot analysis (Supplemental Figure S3C). The presence of smaller C-terminus His6 tagged bands indicates the sensitivity of full-length recombinant TNIP1 to cleavage by endogenous bacterial proteases during expression. To delve further into the potential increased exposure due to intrinsic flexibility, we performed limited proteolysis experiments. Full-length TNIP1 was mixed with limiting trypsin (Figure 2) and chymotrypsin (Supplemental Figure S4B) (both at 1:250, enzyme: TNIP1 (*w*/*w*)) and over 30 min, five separate samples were collected with proteolysis occurring at 25 °C and 37 °C.

Over the time course at both temperatures there is a degradation of the full-length TNIP1 band by 30 min at 25 °C with almost total degradation of all starting protein bands at time zero, aside from one at ~27 kDa, by the 30 min time point. The increased rate of degradation is typical of IDPs, such as characterized IDP alpha-casein [67], especially when compared to globular bovine serum albumin (BSA) (Supplemental Figure S4A). This result indicates the inherent flexibility of TNIP1 and that it is retained in the smaller protein bands yielded during expression and purification.

### 3.3. In Silico Analysis of the Anti-Inflammatory AHD1-UBAN Region of TNIP1

**Figure 3:**
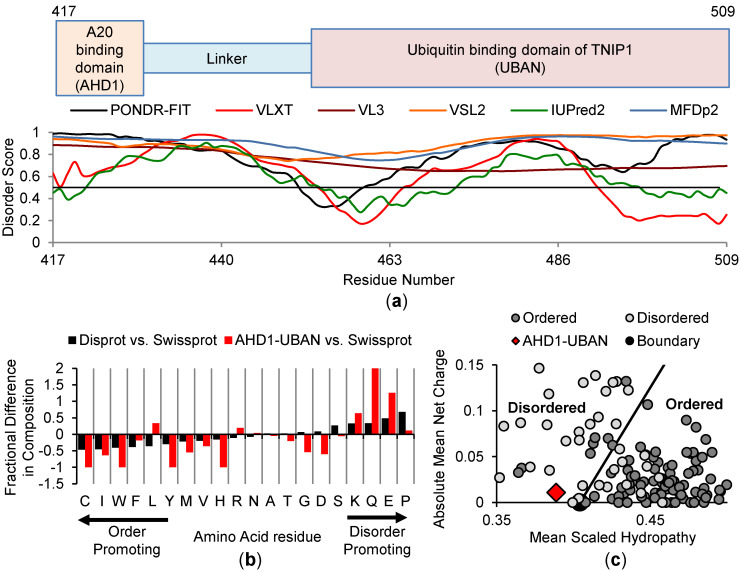
In silico analysis of the TNIP1 AHDI-UBAN domain. (**a**) A20-binding (AHD1) and ubiquitin binding domain of TNIP1 (UBAN) (AA417-509) disorder predictions as generated by independent algorithms where residue disorder score above 0.5 threshold indicates prediction of disorder. (**b**) Amino acid compositional bias of AHD1-UBAN generated comparing AHD1-UBAN versus database of ordered, globular proteins (shown in red) overlaid as comparison against database of disordered proteins (DisProt) versus SwissProt (shown in black). (**c**) Charge–hydropathy plot of AHD1-UBAN (red diamond) with ordered (dark grey circles) and disordered (light grey circles) plotted with boundary representing likely delineation of trends in charge and hydrophobicity between plotted proteins (disordered more likely appearing left of the boundary line).

To investigate the AHD1 and UBAN domain structure, we first took advantage of a similar approach as with the full-length protein, performing in silico analysis of the anti-inflammatory AHD1-UBAN region [25,33,68] with complementary disorder prediction algorithms. Using AA417-509 of full-length TNIP1, we see that outputs of the standalone and meta-predictors show a consensus on most of the TNIP1 AHD1-UBAN construct being disordered (Figure 3a). Current crystal structures of the analogous regions from mouse TNIP1 and human TNIP2 proteins indicate they can achieve a coiled-coiled conformation. Thus we assessed human TNIP1 AHD1-UBAN with DeepCoil (Supplemental Figure S5) [69] and found this region was predicted to form a coiled-coil consistent with the X-ray structures despite the limited amino acid identity (~30% by Clustal Omega) shared by this TNIP1 region and the analogous region in TNIP2. Like the full-length TNIP1 protein, the TNIP1 AHD1-UBAN region shows an amino acid content bias similar to disordered proteins in the Disprot database (as compared to Swissprot) (Figure 3b). There is depletion of hydrophobic core-promoting residues with a great enrichment of polar (glutamine) and charged amino acids (glutamic acid). This is reflected in the charge–hydropathy plot of the AHD1-UBAN region construct (Figure 3c), with this TNIP1 region (red diamond) being located among disordered proteins (light grey circles) left of the boundary line among the increasingly hydrophilic IDPs. Taken together, these different in silico assessments point to the increased likelihood that this region featuring both the AHD1 and UBAN domains are extended and structurally flexible. Additional TNIP1 surface charge factors such as phosphorylation will likely affect its conformation. For instance, D2P2 analysis of TNIP1 reveals several amino acid residues along the full-length protein as sites of possible post-translational modifications (Supplemental Figure S6). In particular, within the AHD1-UBAN AA417-509 region, there are five residues for potential phosphorylation and at least two MoRF regions predicted.

### 3.4. Expression and Limited Proteolysis of the TNIP1 AHD1-UBAN Domain

**Figure 4:**
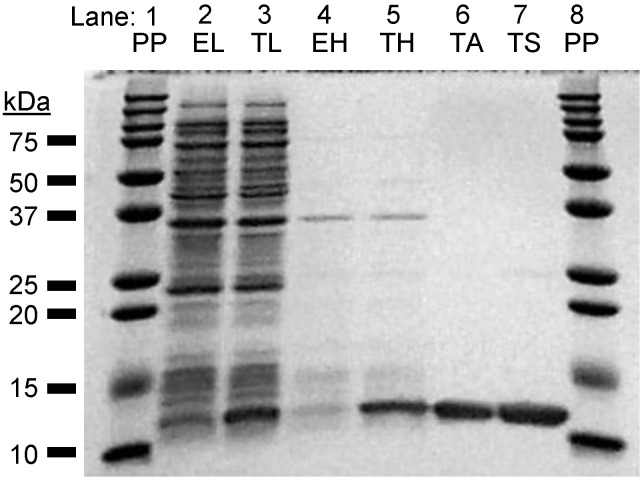
Expression and purification of TNIP1^417-509^. pET28a—empty control (EL) and pET28a-TNIP1^417-509^ (TL) expressed in BL-21 Rosetta 2 *E. coli* released through lysis in Laemmli sample buffer (EL, TL) or by heating to 99 °C (pET28a-empty control heat lysate (EH), pET28a-TNIP1^417-509^ heat lysate (TH)). TNIP1 UBAN fragment purified first by affinity chromatography (TA) followed by FPLC as final purification step in 20 mM sodium phosphate buffer (pH 8.0) and 200 mM NaCl (TS). PP—precision plus pre-stained protein ladder.

Considering peptide regions expressed in structural studies for the analogous regions of human TNIP2 and mouse TNIP1 [21,22], we generated a human TNIP1 AA417-509 expression construct (TNIP1^417-509^) with a C-terminus HIS6 tag (12.7 kDa total predicted molecular weight (MW)). As with the full-length protein work above, solubilization and purification of the TNIP1^417-509^ protein was accomplished with heat lysis of bacterial pellets (Figure 4, TNIP1—lane five) which in multiple reports of recombinant expression of IDPs has proven to promote enrichment of the intact disordered protein and preferential reduction in other heat-denatured proteins (e.g., proteases). The TNIP1^417-509^ resistance to thermal denaturation provided earlier evidence of a non-globular conformation for the region. Perhaps indicating sites for endogenous bacterial proteases outside this region, the TNIP1^417-509^ domain, unlike the full-length TNIP1 protein, had little to no apparent breakdown products. With a combination of affinity purification followed by a final size-exclusion chromatography step (Figure 4—lane seven), the TNIP1^417-509^ was enriched to >95% purity by densitometry analysis of SDS-PAGE resolved samples.

**Figure 5:**
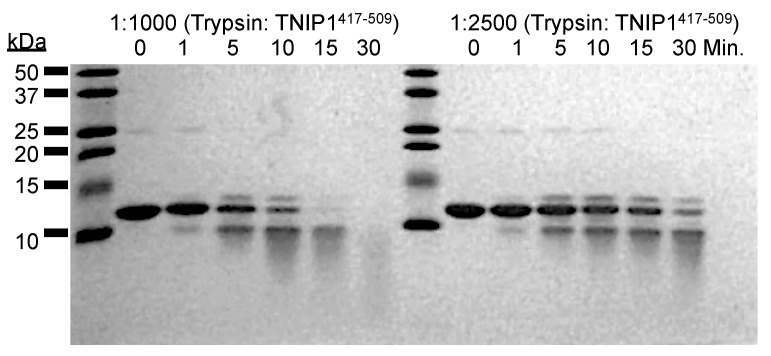
Limited proteolysis of TNIP1^417-509^. Limited proteolysis performed with UBAN fragment with 1:1000 and 1:2500 trypsin to TNIP1^417-509^ protein ratios (*w*/*w*) at 25 °C. Samples collected over 30 min with reactions quenched with addition of 5× Laemmli buffer and heating at 95 °C for 5 min prior to loading 5 μg on a 15% acrylamide gel for analysis by SDS-PAGE.

From in silico analysis showing the TNIP1^417-509^ region as predominantly natively unfolded, we predicted increased in vitro protease sensitivity, paralleling that seen with the full-length protein. To test this, we subjected the expressed region to very limiting amounts of trypsin (trypsin: TNIP1^417-509^ (*w*/*w*) at 1:1000 and 1:2500) at 25 °C (Figure 5). With 1:1000 (trypsin: TNIP1^417-509^), there is no protein remaining at 30 min. As expected with a lower trypsin amount (1:2500 for trypsin: TNIP1^417-509^), there was more intact TNIP1^417-509^ retained with the most obvious difference to the 1:1000 ratio at the 10, 15, and 30 min digestion time points. Taken together, the lack of denaturation during the high heat, thermal-stability lysis and elevated sensitivity to protease at extremely limiting amounts point to the TNIP1^417-509^ construct being natively unfolded with minimal secondary structure and most of the amino acid sequence exposed to the environment.

### 3.5. Analysis of Hydrodynamic and Oligomerization Properties of TNIP1 AHD1-UBAN Region

**Figure 6:**
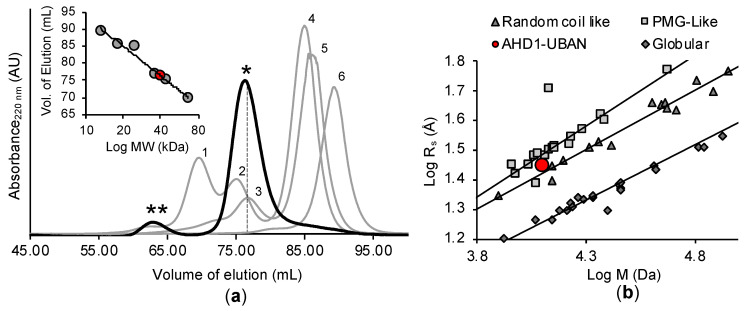
TNIP1^417-509^ domain apparent molecular weight is larger than that predicted from amino acid sequence. Main graph. (**a**) Protein standards (thin lines and numbered peaks) ranging in MW were injected on s75 16/60 high performance column preconditioned with 20 mM sodium phosphate buffer (pH 8.0), 200 mM NaCl: 1—BSA (66 kDa, hydrodynamic radius (Rs)–35 Å); 2—Ovalbumin (44 kDa, Rs–28 Å); 3—Myoglobin dimer (36 Å); 4—Chymotrypsinogen (24 kDa, Rs–21 Å); 5—Myoglobin (18 kDa, Rs–19 Å); 6—Cytochrome C (13 kDa, Rs–17 Å). TNIP1^417-509^ (bold line) eluted as minor (**) and major (*) peaks. Inset graph. Elution volume of protein standards (gray dots) from main graph was plotted against their MW (kDa) and used to determine AHD1-UBAN region apparent MW (red dot). (**b**) Plotted log values of hydrodynamic radii (Å) versus log values of the molecular mass (M) of pre-molten globule like (PMG-like, grey squares), random coil-like (dark grey triangles), and globular proteins (dark grey diamonds). TNIP1^417-509^ (AHD1-UBAN) is presented as a red circle. See Materials and Methods for source of standards used in panel (**b**).

IDP and IDPR frequently display an extended hydrodynamic volume and apparent molecular weight in excess of that predicted from their amino acid content [66]. To assess hydrodynamic radius, we performed analytical gel-filtration with purified TNIP1^417-509^ comparing it against a panel of standards. The TNIP1^417-509^ construct eluted predominantly as one species with a minor, secondary species in an earlier elution fraction (Figure 6a—main graph). Plotting the TNIP1^417-509^ elution volume against the previously run molecular weight standards and their elution volume (Figure 6a—inset graph), showed the TNIP1 AHD1-UBAN region eluted with an apparent molecular weight of ~40 kDa corresponding with a hydrodynamic radius (Rs) of 28.1 Å, greater than the predicted molecular weight (12.7 kDa) and associated hydrodynamic radius (15.4Å) based on encoded amino acid content.

**Figure 7:**
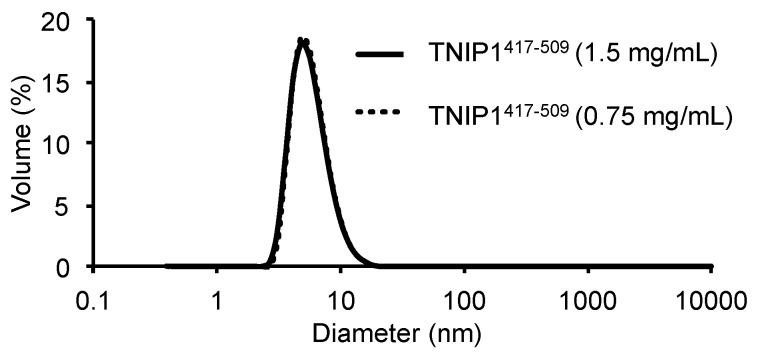
Dynamic light scattering analysis of TNIP1^417-509^. Hydrodynamic radius of AHD1-UBAN fragment measured as percentage of total volume contribution of measurements performed with purified TNIP1^417-509^ in 20 mM sodium phosphate at pH 8.0, 200 mM NaCl, 10% glycerol at two different concentrations (1.5 mg/mL—solid line, 0.75 mg/mL—dotted line).

Comparing the characterized hydrodynamic radius in relation with the molar mass of the TNIP1 AHD1-UBAN to proteins featuring varying degrees of compactness (random coil, pre-molten globule like proteins taken from [53] and globular proteins taken from [54]) (Figure 6b), we see that the TNIP1 AHD1-UBAN domain (red dot) is among the unfolded random coil and pre-molten globule-like proteins. In addition to the prominent species, a minor, secondary species was eluted with an apparent MW of ~100 kDa (apparent hydrodynamic radius of 38 Å), suggesting it may be a self-associated dimer of the TNIP1^417-509^ region. Dynamic light scattering (DLS) of the TNIP1^417-509^ region revealed a moderately polydispersed population (PDI 0.291) measured as total volume by percentage (Figure 7) of the highest of two protein concentrations probed (1.5 mg/mL (117 µM)). One major peak was found for both concentrations probed (1.5 mg/mL (117 µM) and 0.75 mg/mL (58 µM)) with a measured average hydrodynamic radius of 29.6 Å, corresponding with an apparent molecular weight for spherical particles of ~42 kDa. In parallel, molecular weight determination via static light scattering (SLS) reported a MW of 11.3 +/− 1.18 kDa for the sample. This closely matches the monomer weight while being low when compared to the measured hydrodynamic radius, thus suggesting, while featuring some heterogeneity at the concentrations used for the light scattering experiments, that the population consists predominantly of monomeric TNIP1^417-509^. These results parallel and reinforce those from the analytical gel filtration, both data sets pointing to the TNIP1^417-509^ construct being extended and non-spherical in shape with an increased hydrodynamic radius as compared with the predicted and measured MW.

**Figure 8:**
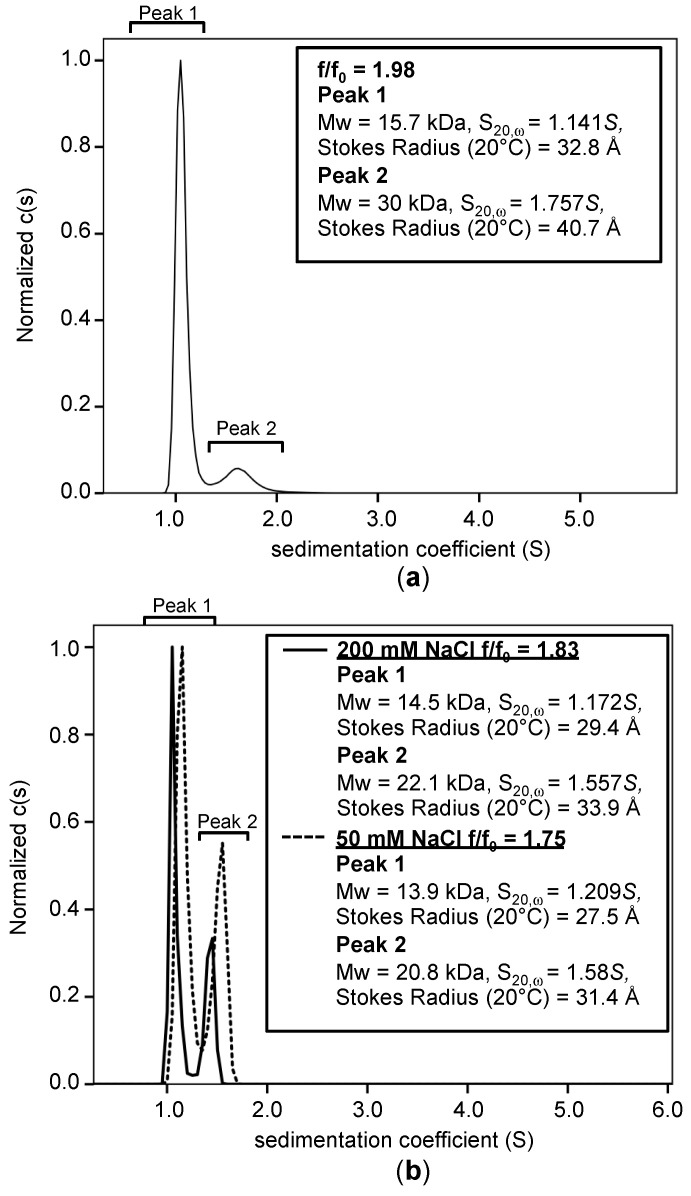
Sedimentation velocity of TNIP1^417-509^ reveals oligomerization potential. Analytical ultracentrifugation sedimentation velocity of purified TNIP1^417-509^ at 20 mM was performed in (**a**) 20 mM sodium phosphate buffer (pH 8.0) and 200 mM NaCl or (**b**) 20 mM sodium phosphate buffer (pH 5.8) and either 50 mM (dotted line) or 200 mM (solid line) NaCl with protein at 20 μM. Experimental runs were performed at 20 °C with scans at 6-min intervals at 230 nm for a total of 20 h. (**a**, **b** insets) Data analysis performed using SedFit.

To combat DLS lacking the resolution needed to parse out the different monomer and oligomer states of the protein, we performed analytical ultracentrifugation (AUC). Sedimentation velocity experiments were conducted using 20 µM (0.25 mg/mL) TNIP1^417-509^ at pH 8.0 (Figure 8a). The observed major species (90% of total) at a sedimentation coefficient (s-value (20, ω) of 1.141*S* had a measured MW of 15.7 kDa (rms residual for fit = 0.0051 fringes), marginally different from the predicted 12.7 kDa. This minor deviation is likely due to the assessed sample consisting of a heterogeneous population. A second species (10% of total) with an s-value (20, ω) of 1.757*S* yielded a measured MW of 30 kDa. This is double that of the major peak and provides distinct biophysical characterization in addition to the above gel filtration and DLS for AHD1-UBAN region dimerization. Stokes radius of the major species was calculated to be 32.8 Å, which closely correlates with the Stokes radii determined by analytical gel filtration and DLS (28.1 and 29.6 Å, respectively). The frictional ratio (f/f_0_), which is indicative of a protein’s shape and used as a primary determining factor of a protein’s intrinsic disorder [70] was determined to be 1.98 (Figure 8a, inset). This value falls within the range that is associated with extended IDPs, whereas globular proteins are closer to 1.2–1.3 [70]. To observe potential pH-dependent oligomerization, we performed AUC at pH 5.8 with 200 mM NaCl (rms residual for fit = 0.0033) and 50 mM NaCl (rms residual for fit = 0.0041) (Figure 8b and summarized in Table 1). We found an increased amount of putative dimer with 200 mM NaCl as compared to the pH 8.0 experimental run (Figure 8), with slightly greater amounts with 50 mM NaCl (pH 5.8, 200 mM NaCl-30%, pH 5.8, 50 mM-38%, pH 8.0, 200 mM NaCl-10%). Interestingly, with increased amounts of what we are calling the dimer peak, we see (Figure 8b, inset) a reduction in the frictional ratio (at 0.25 mg/mL: pH 5.8, 50 mM NaCl-f/f_0_ = 1.75, pH 8.0, 50 mM NaCl-f/f_0_ = 1.98) (Table 1). This may be reflecting increased alpha-helical secondary structure with increased self-association (or compactness) and reduction in the overall intrinsic disorder. These AUC studies indicate TNIP1^417-509^ exists in a mixed population as monomer and dimer, thus supporting the implicated oligomerization from analytical gel filtration and DLS. This dimerization is pH-dependent and is increased under acidic conditions.

### 3.6. Estimation of Secondary Structure within the TNIP1 AHD1-UBAN Region by Circular Dichroism

**Figure 9:**
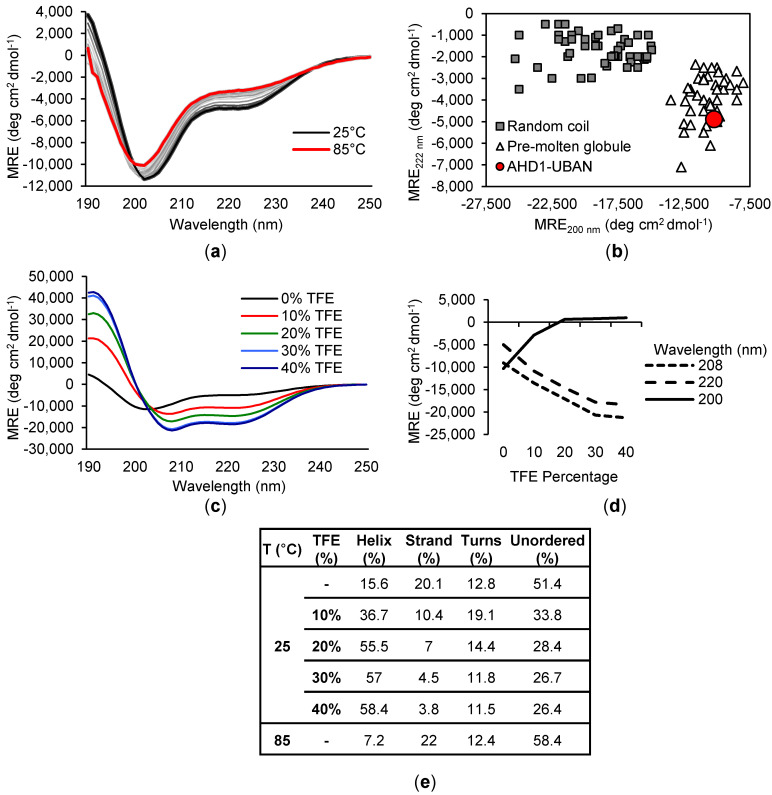
Assessment of TNIP1^417-509^ secondary structure by circular dichroism. Far-UV circular dichroism (CD) spectrum of TNIP1^417-509^ protein at 10 mM in 50 mM sodium phosphate buffer (pH 8.0) scanned from 190–250 nm at (**a**) 25 °C (black line) ramping up to 85 °C (red line). (**b**) Two wavelength plot (222 versus 200 nm) of characterized random coil (grey square) and pre-molten globule (open triangle) like proteins (see Material and Methods) with TNIP1^417-509^ (red circle) grouped among the pre-molten globule group. (**c**) Far-UV CD spectra of TNIP1^417-509^ with increasing titration of 2,2,2-trifluoroethanol (TFE) totaling final 40% (*v*/*v*). (**d**) 208, 220 and 200 nm wavelength measurements from panel B plotted with increasing total TFE percentage. (**e**) Deconvolution of data presented in panel A and C using the Contin-LL (Provencher and Glockner Method) (see Section 2).

To investigate the potential secondary structure of TNIP1^417-509^, we used far-UV circular dichroism (CD) spectroscopy. The obtained far-UV CD spectrum of TNIP1^417-509^ (10 μM) at 25 °C (Figure 9a—black line) shows a common profile associated with IDPs with a characteristic global minima at ~200 nm. However, non-negligible ellipticity between 208 and 222 nm suggests the existence of some secondary structure. Plotting the ellipticity at 200 versus 222 nm (adapted from [45]) (Figure 9b), an analysis method proposed by Uversky for discriminating between random coil and pre-molten globule proteins, shows TNIP1^417-509^ falls in with the pre-molten globule population. Deconvolution of the data using the Contin-LL method [55] reported 56% of the protein structure as unordered.

This percentage disorder is consistent with TNIP1^417-509^ being more pre-molten globule-like, featuring limiting amounts of secondary structure as compared to mostly (or entirely) unfolded random coils [45]. As expected based on the isolation protocol and commonly observed IDP-associated CD spectra, heating the protein from 25 °C to 85 °C (Figure 9a—red line) in five degree steps promoted minimal shift in the overall secondary structure, with a reduction in alpha-helical structure (15.6 to 7.2%) and minor increase in overall unordered content (51.4 to 58.4%). The addition of 2,2,2-trifluoroethanol (TFE), a commonly used co-solvent which promotes alpha-helical structure [71], induced substantial shifts in the far-UV CD spectra of the TNIP1^417-509^ construct (Figure 9c). With increased percent TFE, total alpha-helical content increased inversely as compared to total unordered content. This is apparent with the appearance of a local minimum at 220 nm and shifting of the global minimum from 200 nm to 208 nm (Figure 9d), both characteristic of alpha-helical secondary structure [72]. This is reflected in the deconvolution of the data (Figure 9e) with greater than 58% of the protein consisting of alpha-helices at maximal TFE from 15% alpha-helical content with no TFE. The CD spectra under baseline conditions and with TFE titration indicates that the TNIP1^417-509^ construct is predominantly an unordered pre-molten globule in solution and, like other IDP, has increased alpha-helical features induced by TFE.

### 3.7. ^1^H–^15^N HSQC NMR to Visualize Structure and Dynamics of TNIP1 AHD1-UBAN in Solution

**Figure 10:**
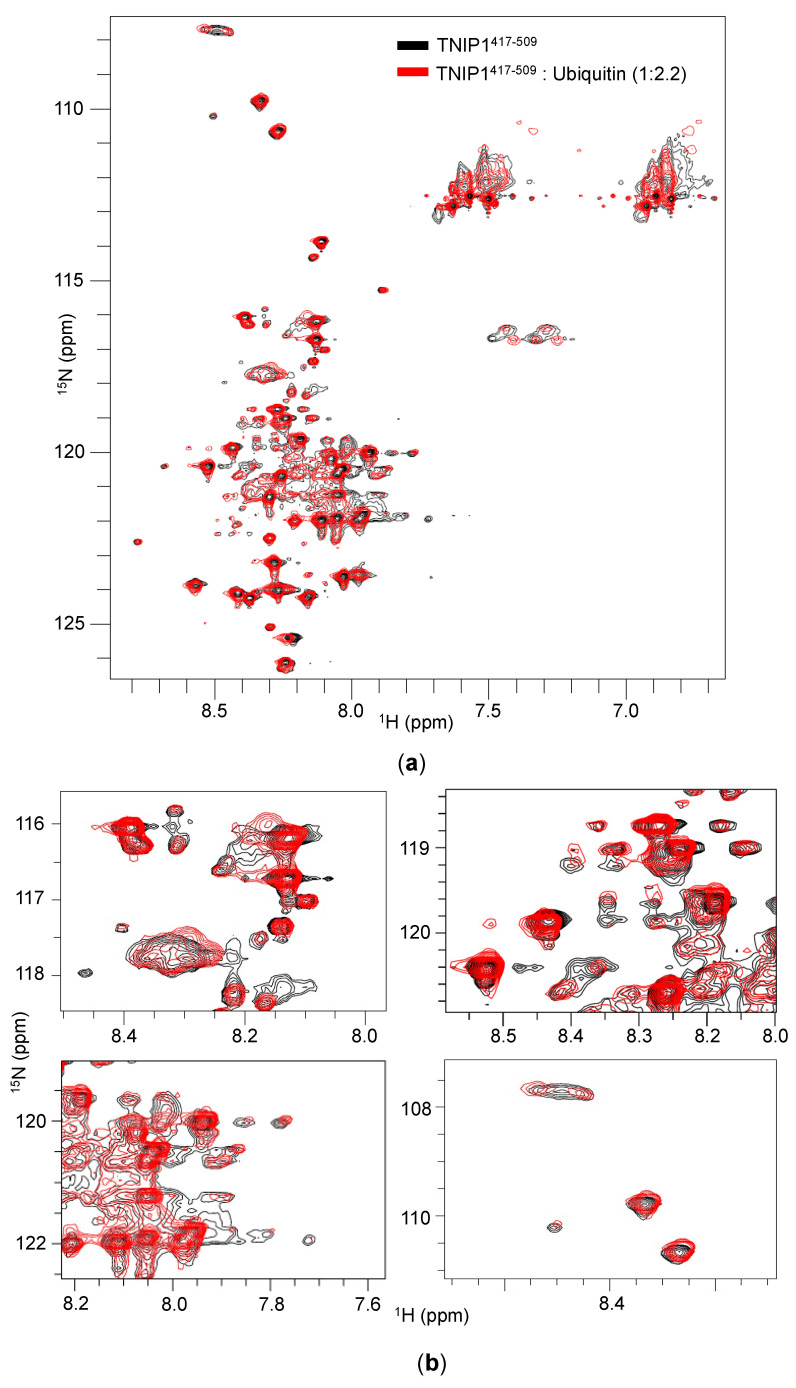
TNIP1 interaction with ubiquitin through NMR. ^1^H-^15^N heteronuclear single quantum coherence (HSQC) spectra at 600 MHz collected for (**a**) TNIP1^417-509^ at 0.3 M only (black) or TNIP1^417-509^ + ubiquitin (red) at a 1:2.2 (TNIP1: ubiquitin) molar ratio at 25 °C in 20 mM sodium phosphate buffer (pH 5.8) and 50 mM NaCl. (**b**) Insets of overlay taken to show details of regions featuring crowding or increased peak shifting with inclusion of ubiquitin.

In the investigation of non-static IDP/IDPR conformations, NMR spectroscopy is a valuable tool providing residue-level information about multiple protein characteristics (e.g., backbone dynamics, overall level of secondary and tertiary structure) simultaneously [73]. We recorded ^1^H–^15^N HSQC 600 MHz NMR spectra for TNIP1^417-509^ alone at pH 6.6 (Supplemental Figure S7A), pH 5.8 (Figure 10a—black and Supplemental Figure S7B) and in complex with ubiquitin [25] (Figure 10a—red). Both HSQC spectra collected at pH 5.8 and 6.6 show poorly dispersed amide regions (8.7–7.8 ppm) [73] with peak proton frequencies situated mainly within the random coil values, a characteristic of disordered proteins (Supplemental Figure S7C). There are fewer amide peaks in the spectrum than expected, due to a number of potential reasons. For instance, lack of amino acid diversity, as reported above (Figure 3b) in our compositional bias analysis, promotes the overlapping of peaks. Peak overlap would be especially furthered in the absence of a folded core which could otherwise provide unique magnetic environments and better dispersion. Additionally, exchange at an intermediate rate (on an NMR timescale) between different conformations, both within the monomeric population and due to intermolecular binding/oligomerization, might result in peaks line broadening beyond detection. The latter is supported in part by the sedimentation velocity experiments performed at pH 5.8, which suggest 38% of the total protein population may exist as a dimer (with 50 mM NaCl).

This is consistent with reduced line broadening in the spectra collected at pH 5.8, potentially revealing beneficial changes in exchange rates associated with increased population of dimerized AHD1-UBAN. This reasoning, however, does not exclude other explanations. For example, sharper peaks may just reflect reduced water–amide exchange. The NMR data further adds that through dimerization, a fixed, static secondary or tertiary structure is not necessarily obtained. However, it remains possible that the putative dimer is invisible being a minor component of the total population, not able to contribute enough detectable secondary or tertiary structure features to be detectible in the spectra. With addition of molar excess amounts of ubiquitin (1:2.2, TNIP1 AHD1-UBAN: ubiquitin), we see a perturbation of resonances (Figure 10b) indicating binding.

However, perturbations are small, suggesting that overall conformational rearrangements do not occur. Several new peaks also appear while others become sharper, suggesting that one of the potential conformations may become predominant/stabilized. Still, the overall spectra remain poorly dispersed suggesting interaction with a binding partner may not facilitate complete adoption of secondary/tertiary structure. Instead, there may be a low affinity interaction that promotes a change in conformational ensemble.

## 4. Discussion

IDP and proteins featuring IDR are a population of proteins which, as compared to globular proteins with fixed conformations, are characterized by dynamic protein backbones, increased polarity/charge, and lack of a tight hydrophobic core. This dynamic, often extended protein structure allows for unique protein conformation assembly through specific modifications (e.g., interaction with binding partners, post-translational modifications). Protein intrinsic disorder is prevalent within proteins critical to the maintenance of human health as regulators of cellular signaling, among other functions [74]. Here, we present work which when taken together positions TNIP1 protein existing as a partially intrinsically disordered protein. Furthermore, isolation, purification and biophysical characterization of the TNIP1 AHD1-UBAN domain (i.e., TNIP1^417-509^) has revealed that it features significant intrinsic disorder. These endpoints were first assessed using bioinformatics methods built on common features among structurally flexible proteins and protein regions (e.g., increased net charge, high hydrophilicity, and enrichment of polar, charged or “structure-breaking” amino acids) [75,76]. We determined that both full-length TNIP1 and TNIP1^417-509^ have amino acid compositions in line with typical IDP and IDR, featuring increased polar and charged residues and deceased occurrence of non-charged residues featuring hydrophobic side chains (Figure 1a and Figure 3b). Consequently, the algorithms used predicted overall increased per-residue disorder scores for both the full-length TNIP1 and TNIP1^417-509^ proteins. This is reflected as well in the predicted disorder agreement from the D2P2 analysis of TNIP1 (Supplementary Figure S6). Included in this analysis are numerous predicted post-translational modification sites, including in the AHD1-UBAN region (Supplementary Figure S6, inset). Phosphoserine position within the local sequence can have either stabilizing or destabilizing consequences for IDR [77] and is an important consideration for TNIP1 in mammalian cells versus the bacterially expressed protein in these recombinant studies. Furthermore, TNIP1 features a phospho-mimetic residue (E470) that corresponds to a critical phosphorylation site in the UBAN of optineurin. Mutation of the E470 residue of TNIP1 promotes a decrease in affinity for polyubiquitin [21]. The expected intrinsically disordered nature of the full-length TNIP1 was found conserved across 23 species (all species had average PONDR-FIT residue scores greater than 0.6 ranging from 0.62–0.71) despite Clustal Omega alignment showing less than 50% sequence identity (Supplemental Table S2B). Conservation of structural disorder in spite of sequence differences is consistent with reports of conservation of protein disorder featuring reduced amino acid homology [78], especially when compared to retention of residue identity in ordered protein domains. This points to the importance of intrinsic disorder within these proteins, even over conserved homology of specific residues.

The recombinant full-length TNIP1 and TNIP1^417-509^ behave as structurally flexible proteins in solution as revealed for both through their high sensitivity to proteolytic digestion (Figure 2 and Figure 5, respectively). Additionally, both the full-length protein and TNIP1^417-509^ region were purified by heat extraction of overexpressed recombinant protein, a method developed for favorably enriching non-globular, non-compaction-dependent proteins. The increased protease sensitivity is likely due to increased solvent accessibility, suggesting both the full-length protein and the TNIP1^417-509^ region are extended and non-compact. This was supported by compatible data from analytical gel filtration and DLS (Figure 6 and Figure 7, respectively) experiments which determined the molecular radius of TNIP1^417-509^ at 28.1 and 29.6 Å, ~1.8× greater than would be expected for a globular form of a protein with similar predicted molecular weight. The analytical gel-filtration and DLS experiments revealed initial data with a potential dimer population of TNIP1^417-509^, which is not unexpected based on reports of the UBAN-only region of murine TNIP1 [21] presenting the UBAN entirely as a coiled-coil. In contrast, our data suggest a mixed population with a secondary peak (Figure 6—double asterisk) from analytical gel-filtration and PDI of 0.291, indicating only moderate polydispersity within our TNIP1^417-509^ samples. Static light scattering determined the molecular weight of the recombinant protein to be 11.3 +/− 1.18 kDa further suggesting the sample is mostly monomeric. For a better-resolved description of the oligomerization state of TNIP1^417-509^, we performed AUC and, at both pH 8.0 and pH 5.8, the major population was near the expected molecular weight of monomer TNIP1^417-509^ (Table 1). There was, however, a second pH sensitive species (ranging from 10 to 38% of the total peak) revealed. This species, approximately double the molecular weight of the major species, is likely dimerized TNIP1^417-509^ as was reported in the murine TNIP1 UBAN crystal structure suggesting the inclusion of the disordered AHD1 and linker present in our AHD1-UBAN region may have affected the extent of the oligomerization. Oligomerization and aggregation of IDPR have gained increased research importance as increasing numbers of IDPR are shown to undergo liquid–liquid phase separation (LLPS) recently reviewed here [79]. Though LLPS is an unknown about TNIP1, it has been reported that when overexpressed in a mammalian cells, TNIP1 forms “aggregates” [4,80] suggesting there may be high level oligomerization occurring at increased concentrations [81,82].

Deconvolution of the far-UV CD spectra of TNIP1^417-509^ (Figure 10) revealed that TNIP1^417-509^ is highly unstructured (56%) in solution. Its secondary structure under basal experimental conditions may also be representative of a mixed population, with the likely more-structured dimer contributing to the overall spectra. Nonetheless, the determined secondary structure was strongly affected with inclusion of the crowding agent TFE, driving the total helical content from 15 to 58%, reducing the overall unstructured component by half. This is in line with this region of TNIP1 having potentially inducible secondary structure as is commonly found in IDPR [83]. Additionally, the CD data suggest that under basal conditions, the protein is mainly unfolded, containing transient secondary structure, i.e., it exists in pre-molten globule form. This becomes especially clear when looking at the analytical gel filtration and CD data (Figure 6b and Figure 9b, respectively) in comparison with characterized IDPs, as was commonly employed in foundational IDP literature [45,53,84,85]. Consistently, the AHD1-UBAN region in solution is grouped among the disordered standards and within the pre-molten globule population. This along with the other data presented here strongly suggests the AHD1-UBAN region features intrinsic disorder when in solution and exists primarily in a monomeric form.

An important feature of intrinsic disorder within protein regions is the concept of continuous shifting between conformational states [86] and existing within a conformational ensemble [87]. The previously discussed data provide an averaged conformational snapshot of TNIP1^417-509^ in solution; protein dynamics are better informed by methods including NMR [87]. The ^1^H–^15^N HSQC NMR spectra of TNIP1^417-509^ (Figure 10a—panel 1) yields poorly dispersed cross peaks with residues mainly in the random coil spectral region, confirming likely protein disorder as determined by the other methods used in this paper. However, a limited number of peaks are resolved and this, while also in support of TNIP1^417-509^ as existing in a dynamic conformational state, is a limit of looking at pre-molten globule-like proteins on the ^1^H–^15^N HSQC NMR timescale. The increased intermediate exchange may be promoted by continuous dimerization of TNIP1^417-509^ monomers or intramolecular exchange through TNIP1^417-509^ existing with a conformational ensemble. However, considering the sedimentation velocity data helps to inform interpretation of NMR spectra as being compatible with some subpopulation of TNIP1^417-509^ being a dimer. We expect the dimer form of TNIP1^417-509^ comprises a minority of the overall species and likely is not the major contributor to dynamic exchange featured in the HSQC spectra. Likely, we are observing TNIP1^417-509^ transitioning between different conformational states as a monomer as well. Future NMR experiments are planned to examine our reasoning. Additionally of note, the more prominent peaks that do not feature any or much line broadening probably represent the most disordered aspect of the protein. The ubiquitin binding studies performed using ^1^H–^15^N HSQC NMR (Figure 10a,b) reveal that the poorly dispersed peaks do not take on a more ordered pattern and indicate the AHD1-UBAN protein does not take on a completely folded structure during such binding. This is not unexpected as the TNIP1 UBAN/monoubiquitin binding has been reported to be a low affinity interaction with a K_D_ of ~100 µM [25]. However, further NMR binding studies need to be performed with increasing lengths of linked-ubiquitin to test whether this holds with polyubiquitin.

The TNIP1 AHD1-UBAN domain, with its role regulating inflammatory cellular signaling, is pictured to function through interaction with multiple partners forming a ternary protein complex [88]. TNIP1 facilitates recruitment and activity of A20 downstream of cell-surface receptor activation for modulation of cytoplasmic signaling [8]. This functionality and the data presented here for the subdomains of the AHD1-UBAN region suggest it, and possibly other TNIP1 regions, function as IDPR effectors, as have been demonstrated in other conformationally adaptable proteins [24]. Furthermore, the data presented here, in concert with previously published UBAN crystal structures, suggest this region features a semi-disordered alpha-MoRF [89] capable of undergoing disorder-to-order transition, a characteristic commonly associated with effector proteins [24]. If this were the case, the intrinsic disorder found within the subdomains of the AHD1-UBAN region may allow for minimal steric hindrance through increased flexibility when contributing to complex assembly triggered by cell signaling activation. Other oligomeric signaling complexes which take advantage of intrinsic disorder have been reported [90,91,92]. Ultimately, the decreased conformational rigidity within the AHD1-UBAN region may promote efficient A20 de-ubiquitination of target proteins by increasing proximity of A20 to polyubiquitin. Crystal structures of the AHD1-UBAN of human TNIP2 [22] have suggested such; however, more research will be required to determine whether this is the case for TNIP1. Related—and also understudied—is the potential role of the linker region flanked by the AHD1 and UBAN of TNIP1. Here, we predicted it to be both highly disordered and structure-breaking. Coincident with this, the linker region lacks predicted coiled-coil formation as it bridges two more-ordered regions (Figure 3a and Supplementary Figure S5). The contribution of this expectedly flexible, proline-rich linker to function within the AHD1-UBAN region will be examined in future studies as it may contribute to proper spatial distancing and independent movement for the AHD1- and UBAN-associated binding motifs [24].

## 5. Conclusions

We employed in silico and in vitro methods in the study of the potential contribution of intrinsic disorder to TNIP1 protein conformation. To investigate algorithm-driven assessments of TNIP1 protein intrinsic disorder, both full-length TNIP1 and the AHD1-UBAN were recombinantly expressed. Both the full-length and AHD1-UBAN construct (TNIP1^417-509^) showed increased structural flexibility with increased sensitivity to protease digestion. The AHD1-UBAN was characterized as having an increased hydrodynamic radius and was determined to mostly exist as a pre-molten globule monomer with inducible secondary structure and oligomerization. This region featuring the AHD1-UBAN domains of TNIP1 is characterized by a dynamic conformation and IDP-like binding characteristics including incomplete adoption of fixed structural order when bound by monoubiquitin. Taken together, the data presented here positions the AHD1-UBAN as a semi-disordered MoRF region within a likely disordered full-length protein which may affect TNIP1 function in inflammatory signal repression.

## Figures and Tables

**Table 1:** Summary of all analytical ultracentrifugation (AUC) experiments. Sedimentation velocity experiments performed across varying pH and salt conditions. Data are summarized from graphs in referenced figures. S, Svedberg unit.

pH	NaCl Concentration	Spectra (Total %)	Protein Concentration	f/f_0_	MW (kDa)	s-Value (20, ω)	Stokes Radius (Å)	Figure
8.0	200 mM	Peak 1 (90%)	0.25 mg/mL (20 μM)	1.98	15.7	1.141*S*	32.8	Figure 8a
		Peak 2 (10%)			30	1.757*S*	40.7	
5.8	200 mM	Peak 1 (70%)	0.25 mg/mL (20 μM)	1.83	14.5	1.172*S*	29.4	Figure 8b (solid line)
		Peak 2 (30%)			22.1	1.557*S*	33.9	
5.8	50 mM	Peak 1 (62%)	0.25 mg/mL (20 μM)	1.75	13.9	1.209*S*	27.5	Figure 8b (dotted line)
		Peak 2 (38%)			20.8	1.582*S*	31.4	

## Supplementary Materials

The following are available online at https://www.mdpi.com/2218-273X/10/11/1531/s1, Figure S1: TNIP1 Clustal Omega multiple sequence alignment. Table S1: Species used in silico assessments of full-length TNIP1. Table S2: Percent identity matrix of full-length TNIP1 species comparisons. Figure S2: Comparison of PONDR-FIT residue scores of TNIP1 species homologs. Figure S3: Expression and purification of full-length TNIP1. Figure S4: Limited proteolysis of full-length TNIP1. Figure S5: Coiled-coil prediction across AHD1 UBAN domains. Figure S6. D2P2 analysis of TNIP1. Figure S7. 1H–15N HSQC spectra of AHD1-UBAN under varying acidic conditions.
