# Supplementary material for: The Anti-Inflammatory Protein TNIP1 Is Intrinsically Disordered with Structural Flexibility Contributed by Its AHD1-UBAN Domain
Source: Biomolecules. 2020 Nov 10;10(11):1531. doi: 10.3390/biom10111531 (PMC7697625; doi:10.3390/biom10111531)
Supplement: Supplementary file 1 [file biomolecules-10-01531-s001.pdf]

CLUSTAL Omega(1.2.4) multiple sequence alignment

```
Homo_sapiens_(Human)                                                                         ---MEGRGPYRIYDPGGSVPSG--E-ASAAFERLVKENSRLKEKMQGIKMLGELLEESQM   54
Mus_musculus_(Mouse)                                                                         ---MEGRGPYRIYDPGGSTPLG--E-VSAAFERLVEENTRLKGKMQGIKMLGELLEESQM   54
Rattus_norvegicus_(Rat)                                                                      ---MEGRGPYRIYDPGGSTPLG--EEVSAAFDRLVEENTRLKGKIQGIKMLGELLEESQM   55
Felis_catus_(Cat)_(Felis_silvestris_catus)                                                   ---MEGRGPYRIYDPGGGVPPG--E-ASAAFERLVEENSRLKEKMQGIKMLGELLEESQM   54
Pan_troglodytes_(Chimpanzee)                                                                 ---MEGRGPYRIYDPGGSVPSG--E-ASTAFERLVKENSRLKEKMQGIKMLGELLEESQM   54
Gallus_gallus_(Chicken)                                                                      MAAMEGRGPYRIYDPGGGTE----ENGSTAFERLIEENARLKERMQGIKSIGELLEESQV   56
Anolis_carolinensis_(Green_anole)_(American_chameleon)                                       ---MEGKGPYRIYDPGGGTK----EEESAALKELLEENIRLKEKMQGMKSLGELLEESQI   53
Monodelphis_domestica_(Gray_short-tailed_opossum)                                            ---MEGRGPYRIYDPGGEGKQG--E-ASAAFEHLVEENSRLKEKMQGIKTLGELLEESQM   54
Ictidomys_tridecemlineatus_(Thirteen-lined_ground_squirrel)_(Spermophilus_tridecemlineatus)  ---MEGRGPYRIYDPGGSVPPG--E-ASAAFERLVEENSRLKEKMQGIKMLGELLEESQM   54
Myotis_lucifugus_(Little_brown_bat)                                                          ---MEGRGPYRIYDPGGSVPPG--E-ASTAFERLVEENSRLKEKMQGIKMLGELLEESQM   54
Otolemur_garnettii_(Small-eared_galago)_(Garnett's_greater_bushbaby)                         ---MEGRGPYRIYDPGGGVPPG--E-ASAAFERLVEENSRLKEKMQGIKMLGELLEESQM   54
Mustela_putorius_furo_(European_domestic_ferret)_(Mustela_furo)                              ---MEGRGPYRIYDPGGGLPLG--E-ASATFERLVEENSRLKEKMQGIKMLGELLEESQM   54
Ficedula_albicollis_(Collared_flycatcher)_(Muscicapa_albicollis)                             ---MEGTGPYHIYDPGGGSE----ENGSTALERLVEENARLKEKMQGIKSIGELLEESQV   53
Chlorocebus_sabaeus_(Green_monkey)_(Cercopithecus_sabaeus)                                   ---MEGRGPYRIYDPGGSVPSG--E-ASTAFERLVKENSRLKEKMQGIKMLGELLEESQM   54
Taeniopygia_guttata_(Zebra_finch)_(Poephila_guttata)                                         ---MEGRGPYHIYDPGGGSE----ENGTTALERLVEENARLKEKMQGIKSIGELLEESQV   53
Pongo_abelii_(Sumatran_orangutan)_(Pongo_pygmaeus_abelii)                                    ---MEGRGPYRIYDPGGSVPSG--E-ASAAFERLVKENSRLKEKMQGIKMLGELLEESQM   54
Macaca_fascicularis_(Crab-eating_macaque)_(Cynomolgus_monkey)_                               ---MEGRGPYRIYDPGGSVPSG--E-ASTAFERLVKENSRLKEKMQGIKMLGELLEESQM   54
Pelodiscus_sinensis_(Chinese_softshell_turtle)_(Trionyx_sinensis)                            ---MEGKGPYRIYDPGGGTQ----EEAAAAFERLVEENTRLKEKMQGIKSLGELLEESQM   53
Bos_taurus_(Bovine)                                                                          ---MEGRGPYRIYDPGGGVPLG--E-ASSAFERLVEENSRLKEKMQGIKMLGELLEESQM   54
Pan_paniscus_(Pygmy_chimpanzee)_(Bonobo)                                                     ---MEGRGPYRIYDPGGSVPSG--E-ASAAFERLVKENSRLKEKMQGIKMLGELLEESQM   54
Capra_hircus_(Goat)                                                                          ---MEGRGPYRIYDPGGGVPLG--E-ASSAFERLVEENSRLKEKMQGIKMLGELLEESQM   54
Danio_rerio_(Zebrafish)_(Brachydanio_rerio)                                                  ---MEGKGPYRIYDPGGSDCQVKDEASSLSYRQLLEENTVLRERMKGLKSLGDLLEESQA   57
Xenopus_tropicalis_(Western_clawed_frog)                                                     ---MESKGPYRIYDPGGSEPSP--S-TNKGVQTLMKDNPALRESMEGIRSLGELLEESEN   54
                                                                                                **. ***:******       .        *:::*  *:  ::*:: :*:*****:

Homo_sapiens_(Human)                                                                         EATRLRQKAEELVKD------NELLPPPSPSLGSFDPLAELTGKDSNVTAS--PTAPA--   104
Mus_musculus_(Mouse)                                                                         EASRLRQKAEELVKDSELS-----PPTSAPSLVSFDDLAELTGQDTKVQVH--PATSTAA   107
Rattus_norvegicus_(Rat)                                                                      EASRLRQKAEALVRDSELP-----PPPPAPSLVSFDPLAELTGQDTKVQAH--PTTTTTT   108
Felis_catus_(Cat)_(Felis_silvestris_catus)                                                   EASRLRQKAEELVKD------SELLPPPSPSLASFDHLAELTGKEADVPAA--PADPA--   104
Pan_troglodytes_(Chimpanzee)                                                                 EATRLRQKAEELVKD------NELLPPPSPSLGSFDPLAELTGKDSNVTAS--PTAPA--   104
Gallus_gallus_(Chicken)                                                                      EASKLRQKAEDLVKDNKMLI----------ASSALEELVETGAAGPE--HSSALAAPGS-   103
Anolis_carolinensis_(Green_anole)_(American_chameleon)                                       EASRLRQKVEDLVQDNDILR----------LTSSLDKLAEIAGEIPDL--CSSSDVPGE-   100
Monodelphis_domestica_(Gray_short-tailed_opossum)                                            EASKLRQKVEELAKDNEL-------LQPSPPSSSFDHLAELSGGNTNLELP--PVNPAGS   105
Ictidomys_tridecemlineatus_(Thirteen-lined_ground_squirrel)_(Spermophilus_tridecemlineatus)  EASRLRQKAEELVKDNE-------PHPPCPSLSSFDHLTELTGEDTNTPAP--PVAPA--   103
Myotis_lucifugus_(Little_brown_bat)                                                          EASRLRQKAEALVQDSELGKDRQLLPPPSPTLTSFDHLPELTGKDANVPAP--PADPA--   110
Otolemur_garnettii_(Small-eared_galago)_(Garnett's_greater_bushbaby)                         EASRLRQKAEELVKD------NEPLPPPSPSLGSFDQLAELTGTDPNVPAT--PTAPT--   104
Mustela_putorius_furo_(European_domestic_ferret)_(Mustela_furo)                              EASRLRQKAEELVKD------SELLPTPSPSLASYDHLAELARKDAGGPAP--PADPA--   104
Ficedula_albicollis_(Collared_flycatcher)_(Muscicapa_albicollis)                             EASKLRQKAEDLVKDNKMLI----------GSSSLENLVETGAVGPD--PSFARAAPGS-   100
Chlorocebus_sabaeus_(Green_monkey)_(Cercopithecus_sabaeus)                                   EATRLRQKAEELVKD------NELLPPPSPSLGSFDPLAELTGKDSNVTAP--PTAPA--   104
Taeniopygia_guttata_(Zebra_finch)_(Poephila_guttata)                                         EASKLRQKAEDLVKDNKMLI----------GSSSLEDLNPELEGRERLEVCSPKAHGCI-   102
Pongo_abelii_(Sumatran_orangutan)_(Pongo_pygmaeus_abelii)                                    EATRLRQKAEELVKD------NELLPPPSPSLGSFDPLAELTGKDSNVTAP--PTAPA--   104
Macaca_fascicularis_(Crab-eating_macaque)_(Cynomolgus_monkey)_                               EATRLRQKAEELVKD------NELLPPPSPSLGSFDPLAELTGKDSNVTAP--PTAPA--   104
Pelodiscus_sinensis_(Chinese_softshell_turtle)_(Trionyx_sinensis)                            EASKLRQKVEDLVKDNEMLR----------S-SSFDKLAEITGGDAE--PNQSPTGPGK-   99
Bos_taurus_(Bovine)                                                                          EASRLRQKAEELVKD------SEPLPPSSPSLASFNDLAELAGKDTAVPAT--PADPA--   104
Pan_paniscus_(Pygmy_chimpanzee)_(Bonobo)                                                     EATRLRQKAEELVKD------NELLPPPSPSLGSFDPLAELTGKDSNVTAS--PTAPA--   104
Capra_hircus_(Goat)                                                                          EASRLRQKAEELVKD------SEPLPPPSPSLASFNNLAELAGKDTAVPAT--PADPA--   104
Danio_rerio_(Zebrafish)_(Brachydanio_rerio)                                                  EAAKLRKRVEELVRDNEVLKSSATSFASSLCMGTP-VQTETHGHGKRH------GHPT--   108
Xenopus_tropicalis_(Western_clawed_frog)                                                     EVYRLRQKAEALVKDNKHLHSSSAMDSQ----PSFL---EETVLGRKLSAGSEQAEGLQ-   106
                                                                                             *. :**::.* *.:*                  :

Homo_sapiens_(Human)                                                                         ----------CPSDKPAPVQKPPSSGTSSEFEVVTPEEQNS-PESSSH-ANAMALG--PL   150
Mus_musculus_(Mouse)                                                                         T-TTATATTGNSMEKPEPASKSPSNGASSDFEVVPTEEQNS-PETGSHPTNMMDLG--PP   163
Rattus_norvegicus_(Rat)                                                                      TTTTTTTTIGNSMEKSVSASKPPSNGASSDFEVVPPEEQSS-PETGHHSRNVMELG--SP   165
Felis_catus_(Cat)_(Felis_silvestris_catus)                                                   ----------HPSNKAEPVQKPPSSGTSSEFEVVPAEEQNSPPASGGQARNTMELG--PL   152
Pan_troglodytes_(Chimpanzee)                                                                 ----------CPSDKPAPVQKPPSSGTSSEFEVVTPEEQNS-PESSSH-ANAMALG--PL   150
Gallus_gallus_(Chicken)                                                                      ----------AQPD---TGTQKSPSGSSSEFEVVAAEAQ-GCPQDSGRAEL----E--PL   143
Anolis_carolinensis_(Green_anole)_(American_chameleon)                                       ----------NQKDMEKATPKPSSSGSSSEFEIVGAEDKRSFPDNSRKPGI----E--PV   144
Monodelphis_domestica_(Gray_short-tailed_opossum)                                            -----------RNEQAPTVQKTPSSGASSEFEVVSPEEHKSPS-GGRGNSDMLDLT--PL   151
Ictidomys_tridecemlineatus_(Thirteen-lined_ground_squirrel)_(Spermophilus_tridecemlineatus)  ----------CPSDKSASAPKPPSSGTSSEFEIVHTEEQNSPPESRGHIRNPMELG--PL   151
Myotis_lucifugus_(Little_brown_bat)                                                          ----------PPSDKPEPVQKPPSSGTSSEFEVVP-NEQNSPPESGGQTRNMMELG--PL   157
Otolemur_garnettii_(Small-eared_galago)_(Garnett's_greater_bushbaby)                         ----------CPSDKPAPAQKPPSSGTSSEFEVVPTEEQNSPPESGGR-TNTMELG--PL   151
Mustela_putorius_furo_(European_domestic_ferret)_(Mustela_furo)                              ----------HPSDKPEPVQKPPSSGTSSEFEVVPADDQTSPPASGGHARNTKVLG--PL   152
Ficedula_albicollis_(Collared_flycatcher)_(Muscicapa_albicollis)                             ----------AQPD---LEARKSPP----------------------SDL----E--QP   118
Chlorocebus_sabaeus_(Green_monkey)_(Cercopithecus_sabaeus)                                   ----------CPSDKPAPVQKPPSSGTSSEFEVVTPEEQSSPPESSSH-ANETVLG--PL   151
Taeniopygia_guttata_(Zebra_finch)_(Poephila_guttata)                                         ----------LSPL--HSPRKSPPSGSSSEFEIVAIEAQ-GFPQEGRRAEN----MGIGP   145
Pongo_abelii_(Sumatran_orangutan)_(Pongo_pygmaeus_abelii)                                    ----------CPSDKPAPVQKPPSSGTSSEFEVVTPEEQNS-PESSSH-ANAMALG--PL   150
Macaca_fascicularis_(Crab-eating_macaque)_(Cynomolgus_monkey)_                               ----------CPSDKPAPVQKPPSSGTSSEFEVVTPEEQSSPPENSSH-ANETLLG--PL   151
Pelodiscus_sinensis_(Chinese_softshell_turtle)_(Trionyx_sinensis)                            ----------TEKQQDTQAQKPPSTGSSSEFEIVSSEDK-RFSQESRKSDHLLTAPEVEL   148
Bos_taurus_(Bovine)                                                                          ----------HPSDKPEPVPKPPSSNTSSEFEVVPTEGP-S-PESSGHTRNTMELG--PL   150
Pan_paniscus_(Pygmy_chimpanzee)_(Bonobo)                                                     ----------CPSDKPAPVQKPPSSGTSSEFEVVTPEEQNS-PESSSH-ANAMALG--PL   150
Capra_hircus_(Goat)                                                                          ----------HPSNKPEPVPKPPSSNASSEFEVVPAEGP-SSPESSGHTRNTMELG--PL   151
Danio_rerio_(Zebrafish)_(Brachydanio_rerio)                                                  -----TERQESRDCLTGNTLQQEATEGSSEFEVVNMEEKTAPET--------QTAGVLHL   155
Xenopus_tropicalis_(Western_clawed_frog)                                                     --SPKM-TDLDSDVHPKSNQKTPPSGTSSEFEILNAYETRKTPESRKLD------PGPTL   157
                                                                                                                 :

Homo_sapiens_(Human)                                                                         PREDGNLMLHLQRLETTLSVCAEEPDHGQLFTHLGRMALEFNRLASKVHKNEQRTSILQT   210
Mus_musculus_(Mouse)                                                                         PPEDSNLKLHLQRLETTLSVCAEEPDHSQLFTHLGRMALEFNRLASKVHKNEQRTSILQT   223
Rattus_norvegicus_(Rat)                                                                      PPEDGNLMLHLQRLETTLSVCAEEPDHSQLFTHLGRMALEFNRLASKVHKNEQRTSILQT   225
Felis_catus_(Cat)_(Felis_silvestris_catus)                                                   PHEDTNLMLHLQRLETTLSVCAEEPDHSQLFTHLGRMALEFNRLASKVHKNEQRTSILQT   212
Pan_troglodytes_(Chimpanzee)                                                                 PHEDGNLMLHLQRLETTLSVCAEEPDHGQLFTHLGRMALEFNRLASKVHKNEQRTSILQT   210
Gallus_gallus_(Chicken)                                                                      PHEDANLLPQLQQLESTLSGCAREASKDQVFVRMGYMASELKRLASKVHKNEQRTSFLQT   203
Anolis_carolinensis_(Green_anole)_(American_chameleon)                                       PNEDANLMLHLQRLENTLSLFAEESDKTQLFAHLGRMALEFNRLASKVHKNEQRTSILQA   204
Monodelphis_domestica_(Gray_short-tailed_opossum)                                            PKEDSNLMLHLQRLESTLSVCAEEPDHTQVFTHLGRMALEFNRLASKVHKNEQRTSILQT   211
Ictidomys_tridecemlineatus_(Thirteen-lined_ground_squirrel)_(Spermophilus_tridecemlineatus)  PHEDSNLMLHLQRLETTLSVCAEEPDHSQLFTHLGRMALEFNRLASKVHKNEQRTSILQT   211
Myotis_lucifugus_(Little_brown_bat)                                                          PHEDSNLMLHLQRLETTLSVCAEEPDHSQLFTHLGRMALEFNRLASKVHKNEQRTSILQT   217
Otolemur_garnettii_(Small-eared_galago)_(Garnett's_greater_bushbaby)                         PHEDSNLMLHLQRLETTLSVCAEEPDHSQLFTHLGRMALEFNRLASKVHKNEQRTSILQT   211
Mustela_putorius_furo_(European_domestic_ferret)_(Mustela_furo)                              PHEDSNLMLHLQRLESTLSVCAEEPDHSQLFTHLGRMALEFNRLASKVHKNEQRTSILQT   212
Ficedula_albicollis_(Collared_flycatcher)_(Muscicapa_albicollis)                             PNEDANLLPQLQQLENTLSGCAKEASKDQVFVRMGYMASELKRLASKVHKNEQRTSFLQT   178
Chlorocebus_sabaeus_(Green_monkey)_(Cercopithecus_sabaeus)                                   PHEDSNLMLHLQRLETTLSVCAEEPDHGQLFTHLGRMALEFNRLASKVHKNEQRTSILQT   211
Taeniopygia_guttata_(Zebra_finch)_(Poephila_guttata)                                         LNEDANLLPQLQQLENTLSGCAKEASKDQVFVRMGYMASELKRLASKVHKNEQRTSFLQT   205
Pongo_abelii_(Sumatran_orangutan)_(Pongo_pygmaeus_abelii)                                    PHEDGNLMLHLQRLETTLSVCAEEPDHSQLFTHLGRMALEFNRLASKVHKNEQRTSILQT   210
Macaca_fascicularis_(Crab-eating_macaque)_(Cynomolgus_monkey)_                               PHEDGNLMLHLQRLETTLSVCAEEPDHSQLFTHLGRMALEFNRLASKVHKNEQRTSILQT   211
Pelodiscus_sinensis_(Chinese_softshell_turtle)_(Trionyx_sinensis)                            QNEDTNLMLHLQRLESTLSVFAEEPDKNQVLAHLGRMALEFNRLASKVHKNEQRTSILQT   208
Bos_taurus_(Bovine)                                                                          PQEDSTLMLHLQRLETTLSVCAEEPDHSQLFTHLGRMALEFNRLASKVHKNEQRTSILQT   210
Pan_paniscus_(Pygmy_chimpanzee)_(Bonobo)                                                     PHEDGNLMLHLQRLETTLSVCAEEPDHGQLFTHLGRMALEFNRLASKVHKNEQRTSILQT   210
Capra_hircus_(Goat)                                                                          PQEDSTLMLHLQRLETTLSVCAEEPDHSQLFTHLGRMALEFNRLASKVHKNEQRTSILQT   211
Danio_rerio_(Zebrafish)_(Brachydanio_rerio)                                                  PQENLELASQLRRLESSFSVFAEESNPNQLLAHLGRMAVEFHHLSSKVQKNEQRTSLLQT   215
Xenopus_tropicalis_(Western_clawed_frog)                                                     PQEDDNVQLHLQRMETSLSMFAEEPDRKSPIAHISRLALDFSRLASKVHKNEQKTAVLQT   217
                                                                                               *:  :  :*:::*.::*  *.* .  . :.::. :* :: :*:***:****:*:.**:
```

```
Homo_sapiens_(Human)                                                                    LCEQLRKENEALKAKLDKGLEQRDQAAERLREENLELKKLLMSN-GNKEGASGRPGSPKM     269
Mus_musculus_(Mouse)                                                                    LCEQLRQENEALKAKLDKGLEQRDLAAERLREENTELKKLLMNS-SCKEGLCGQPSSPKP     282
Rattus_norvegicus_(Rat)                                                                 LCEQLRQENEALKAKLDKSLEQRDQAAERLREENTELKRLLMNS-NCKEGLCGQPSSPKT     284
Felis_catus_(Cat)_(Felis_silvestris_catus)                                              LCEQLRKENEALKAKLDKGLEQRDQAAERLREENMELKKLLMSC-GK-EDTCGQPGFPKM     270
Pan_troglodytes_(Chimpanzee)                                                            LCEQLRKENEALKAKLDKGLEQRDQAAERLREENLELKKLLMSN-GNKEGASGRPGSPKM     269
Gallus_gallus_(Chicken)                                                                 LCETLHTENKELRTKLERDLEQRNQALEKLRCENQELRRMVTLSN--QESAKREAAE---     258
Anolis_carolinensis_(Green_anole)_(American_chameleon)                                  LCEQLRNENEELRTKLEKELEQRNQISEKLRCENLELRKMVMMGN--KEGMKAESTEF--     260
Monodelphis_domestica_(Gray_short-tailed_opossum)                                       LCEQLRNENEALKAKLEKDLEQRDQATEKLRQENLELKKLVMSGSG-KEGATGLPGGLRM     270
Ictidomys_tridecemlineatus_(Thirteen-lined_ground_squirrel)_(Spermophilus_tridecemlineatus)   LCEQLRKENEALKAKLDKGLEQRDQAAERLREENMELKKLLMSSSSNKESACGQPGSPKL     271
Myotis_lucifugus_(Little_brown_bat)                                                     LCEQLRKENETLKSKLDKGLEQRDQAAERLREENMELKKLLMTS-GK-EGVCRRPGSPKM     275
Otolemur_garnettii_(Small-eared_galago)_(Garnett's_greater_bushbaby)                    LCEQLRKENEALKAKLDKGLEQRDQAAERLREENMELKKLLMSS-GNKEGASGWPLSPKM     270
Mustela_putorius_furo_(European_domestic_ferret)_(Mustela_furo)                         LCEQLRKENEALKAKLDKGLEQRDQAAERLREENLELKKLLMSC-GK-EDTCGQPGSPKT     270
Ficedula_albicollis_(Collared_flycatcher)_(Muscicapa_albicollis)                        LCETLHNENKELRTKLEHDLEQRNQALEKLRCENQELRRMVTLSS--QDSGKREAAE---     233
Chlorocebus_sabaeus_(Green_monkey)_(Cercopithecus_sabaeus)                              LCEQLRKEKEALKAKLDKGLEQRDQAAERLREENLELKKLLMSN-GNKEGASGQPGSPKM     270
Taeniopygia_guttata_(Zebra_finch)_(Poephila_guttata)                                    LCETLHSENKELRTKLEHDLEQR-QALEKLRCENQELRRMVTLSS--QDSGKGKPPSSSR     262
Pongo_abelii_(Sumatran_orangutan)_(Pongo_pygmaeus_abelii)                               LCEQLRKENEALKAKLDKGLEQRDQAAERL------------------------------     240
Macaca_fascicularis_(Crab-eating_macaque)_(Cynomolgus_monkey)_                          LCEQLRKENEALKAKLDKGLEQRDQAAERLREENLELKKLLMSN-GNKEGASGQPGSLKM     270
Pelodiscus_sinensis_(Chinese_softshell_turtle)_(Trionyx_sinensis)                       LCEQLRNENEDLRAKLEKDLEQRNQAMEKLRCENLELRKMVMSSK--A-GVK--------     257
Bos_taurus_(Bovine)                                                                     LCEQLRKENEALKSKLDKGLEQRDQAAERLREENLELRKLLMSSS--KDGASGRPGSPKM     268
Pan_paniscus_(Pygmy_chimpanzee)_(Bonobo)                                                LCEQLRKENEALKAKLDKGLEQRDQAAERLREENLELKKLLMSN-GNKEGASGRPGSPKM     269
Capra_hircus_(Goat)                                                                     LCEQLRKENEALKSKLDKGLEQRDQAAERLREENLELRKLLMSSS--KDGASGRPGSPKM     269
Danio_rerio_(Zebrafish)_(Brachydanio_rerio)                                             LCEQLRQENSELRKKMEDDLQYRNRDLEQLRQENLKLKEQVSGASHT---AHVEQLNPKE     272
Xenopus_tropicalis_(Western_clawed_frog)                                                LCEQLRKENEDLRAKLEADWNQRTQAEQALRYENLELKKLILQSD--RGGGKGFHAGGGV     275
                                                                                        *** *: *:. *: *::    : * : *

Homo_sapiens_(Human)                                                                    EGTG------KKAVAGQQQASVTAGKVPEVV-ALGAAEKKVKMLEQQRSELLEVNKQWDQ     322
Mus_musculus_(Mouse)                                                                    EGAG------KKGVAGQQQASVMASKVPEAG-AFGAAEKKVKLLEQQRMELLEVNKQWDQ     335
Rattus_norvegicus_(Rat)                                                                 EGAG------KKGVAGQQQASMTASKVPEAG-AFGTAEKKVKLLEQQRMELLEVNKQWDQ     337
Felis_catus_(Cat)_(Felis_silvestris_catus)                                              EGAG------KKGVAGQQQACVTAGKIPEVG-ALGAAEKKVKMLEQQRAELLEVNKQWDQ     323
Pan_troglodytes_(Chimpanzee)                                                            EGTG------KKAVAGQQQASVTASKVPEVV-ALGAAEKKVKMLEQQRSELLEVNKQWDQ     322
Gallus_gallus_(Chicken)                                                                 --------------QQQQSGVVVEKAAGRG-ELEAKEKKVKILEHQRRELLEVNKQWDQ     302
Anolis_carolinensis_(Green_anole)_(American_chameleon)                                  -------------AGQQQKGAAEEKVTVKS-MLGTSEKKVKILEHQRNELLEVNKQWDQ     305
Monodelphis_domestica_(Gray_short-tailed_opossum)                                       EVSG------RKSLARQQQAGMSPGNIPEAG-TMGATEKKVKILEQQRSELLEVNKQWDQ     323
Ictidomys_tridecemlineatus_(Thirteen-lined_ground_squirrel)_(Spermophilus_tridecemlineatus)   EGNS------KKGMAGQQQTGVMAGKVPEVG-ALGAAEKKVKMLEQQRMELLEVNKQWDQ     324
Myotis_lucifugus_(Little_brown_bat)                                                     EGAG------KKGVAGQLQASATASKIPEAG-ALGAAEKKVKMLEQQRTELLEVNKQWDQ     328
Otolemur_garnettii_(Small-eared_galago)_(Garnett's_greater_bushbaby)                    EGAG------KKEVAGQQQASMTAGKGPEVG-ALGAAEKKVKMLEQQRTELLEVNKQWDQ     323
Mustela_putorius_furo_(European_domestic_ferret)_(Mustela_furo)                         EGVG------KKGVAGHQQACAAAGKSPEVG-ALGAAEKKVKMLEQQRTELLEVNKQWDQ     323
Ficedula_albicollis_(Collared_flycatcher)_(Muscicapa_albicollis)                        --------------Q-QQSGAMVEKAPGRE-ELEAKEKKVKILEHQRRELLEVNKQWDQ     276
Chlorocebus_sabaeus_(Green_monkey)_(Cercopithecus_sabaeus)                              EGAG------KKAVAGQQQASVTAGKVPEVA-ALGAAEKKVKMLEQQRSELLEVNKQWDQ     323
Taeniopygia_guttata_(Zebra_finch)_(Poephila_guttata)                                    TSQG------LKG-MGEAQSGAMVEKAPGRE-ELEAKEKKVKILEHQRRERKRCREVWGL     314
Pongo_abelii_(Sumatran_orangutan)_(Pongo_pygmaeus_abelii)                               ------------------ASVTAGKVPEVA-ALGAAEKKVKMLEQQRSELLEVNKQWDQ     280
Macaca_fascicularis_(Crab-eating_macaque)_(Cynomolgus_monkey)_                          EGAG------KKAVAGQQQASVTAGKVPEVA-ALGAAEKKVKMLEQQRSELLEVNKQWDQ     323
Pelodiscus_sinensis_(Chinese_softshell_turtle)_(Trionyx_sinensis)                       -GEG------VES-AGQQQSGAPAEKAAGKG-TAGASEKKVKILEHQRNELLEVNKQWDQ     308
Bos_taurus_(Bovine)                                                                     EGAG------KKGAAAPQQAGVTTSKIPEVG-SLGAAEKKVRMLEQQRTELLEVNKQWDQ     321
Pan_paniscus_(Pygmy_chimpanzee)_(Bonobo)                                                EGTG------KKAVAGQQQASVTASKVPEVV-ALGAAEKKVKMLEQQRSELLEVNKQWDQ     322
Capra_hircus_(Goat)                                                                     EGTG------KKGAAALQQAGVTASKTPEVG-PLGAAEKKVRMLEQQRTEVGG---RILP     319
Danio_rerio_(Zebrafish)_(Brachydanio_rerio)                                             EPVKEEAVKTKVEMTMTQQSGKAVEKTPSKTCDPEMYEKKIKLLEKQRKDVLEVNKQWDI     332
Xenopus_tropicalis_(Western_clawed_frog)                                                DSAAQE-DAVKLENPGIQQVAKLMEKPAVKD-PSVSLLKKVKILEHQRTELLEVNKQWDQ     333
                                                                                                                   :             **:::**:** :

Homo_sapiens_(Human)                                                                    HFRSMK--QQYEQKITELRQKLADLQKQVTDLEAEREQKQRDFDRKLLLAKSKIEMEETD     380
Mus_musculus_(Mouse)                                                                    HFRSMK--QQYEQKITELRQKLVDLQKQVTELEAEREQKQRDFDRKLLLAKSKIEMEETD     393
Rattus_norvegicus_(Rat)                                                                 HFRTMK--QQYEQKITELRQKLVDLQKQVTELEAEREQKQRDFDRKLLLAKSKIEMEETD     395
Felis_catus_(Cat)_(Felis_silvestris_catus)                                              HFRSMK--QQYEQKITELRQKLADLQKQVTDLEAEREQKQRDFDRKLLLAKSKIEMEETD     381
Pan_troglodytes_(Chimpanzee)                                                            HFRSMK--QQYEQKITELRQKLADLQKQVTDLEAEREQKQRDFDRKLLLAKSKIEMEETD     380
Gallus_gallus_(Chicken)                                                                 HFRAMK--QKYEQKMTDLHQELAEARRALTELESEREQKQRDFDRKLLLAKSRIETEEAE     360
Anolis_carolinensis_(Green_anole)_(American_chameleon)                                  HFRSMK--QQYEQKITDLRQKLADSQKAGGEMEAEREQKQRDFDRKLLLAKSKIEEEEAE     363
Monodelphis_domestica_(Gray_short-tailed_opossum)                                       HFRSMK--QQYEQKITELRQKLADSQKLVSELESDREQKQRDFDRKLLLAKSKIEMEEAD     381
Ictidomys_tridecemlineatus_(Thirteen-lined_ground_squirrel)_(Spermophilus_tridecemlineatus)   HFRSMK--QQYEQKITELRQKLADLQKQVTDLEAEREQKQRDFDRKLLLAKSKIEMEENN     382
Myotis_lucifugus_(Little_brown_bat)                                                     HFRSMK--QQYEQKITELRQKLADLQKQVTDLEAEREQKQRDFDRKLLLAKSKIEMEETD     386
Otolemur_garnettii_(Small-eared_galago)_(Garnett's_greater_bushbaby)                    HFRSMK--QQYEQKITELRQKLADLQKQVTELEAEREQKQRDFDRKLLLAKSKIEMEETD     381
Mustela_putorius_furo_(European_domestic_ferret)_(Mustela_furo)                         HFRSMK--QQYEQKITELRQKLADFQKQVTDLEAEREQKQRDFDRKLLLAKSKIEMEETD     381
Ficedula_albicollis_(Collared_flycatcher)_(Muscicapa_albicollis)                        HFRSMK--QKYEQKVTDLHQELAEARRAVTELESEREQKQRDFDRKLLLAKSRIETEEAE     334
Chlorocebus_sabaeus_(Green_monkey)_(Cercopithecus_sabaeus)                              HFRSMK--QQYEQKITELRQKLADLQKQVTDLEAEREQKQRDFDRKLLLAKSKIEMEETD     381
Taeniopygia_guttata_(Zebra_finch)_(Poephila_guttata)                                    PVPPFPQRGSFQANVTDLHQELAEARRAVTELESEREQKQRDFDRKLLLAKSRIETEEAK     374
Pongo_abelii_(Sumatran_orangutan)_(Pongo_pygmaeus_abelii)                               HFRSMK--QQYEQKITELRQKLADLQKQVTDLEAEREQKQRDFDRKLLLAKSKIEMEETD     338
Macaca_fascicularis_(Crab-eating_macaque)_(Cynomolgus_monkey)_                          HFRSMK--QQYEQKITELRQKLADLQKQVTDLEAEREQKQRDFDRKLLLAKSKIEMEETD     381
Pelodiscus_sinensis_(Chinese_softshell_turtle)_(Trionyx_sinensis)                       HFRTMK--QQYEQKITDLRQKLADSQRALSEQEAEREQKQRDFDRKLLLAKSKIEVEEAD     366
Bos_taurus_(Bovine)                                                                     HFRSMK--QQYEQKITELRQKLADLQKQVTDLEAEREQKQRDFDRKLLLAKSKIEMEETD     379
Pan_paniscus_(Pygmy_chimpanzee)_(Bonobo)                                                HFRSMK--QQYEQKITELRQKLADLQKQVTDLEAEREQKQRDFDRKLLLAKSKIEMEETD     380
Capra_hircus_(Goat)                                                                     HFRGL---------------QHKDLQKQVTDLEAEREQKQRDFDRKLLLAKSKIEMEETD     364
Danio_rerio_(Zebrafish)_(Brachydanio_rerio)                                             QWNSMK--TQFEQKITDLRQRLADSQKTVLELEAEREQRQRDYDKKLLLAKSKIDNVQGE     390
Xenopus_tropicalis_(Western_clawed_frog)                                                QFRSMK--MQYEEKITSLRQKLSQASKAESEQEVEKDRKQRDFDRKLLLARDKIEEKESQ     391
                                                                                            :                .  :  :    : * :::::***:*:*****:.:*:  :  .

Homo_sapiens_(Human)                                                                    KEQLTAEAKELRQKVKYLQDQLSPLTRQREYQEKEIQRLNKALEEALSIQTPPSSPPTAF     440
Mus_musculus_(Mouse)                                                                    KEQLTAEAKELRQKVRYLQDQLSPLTRQREYQEKEIQRLNKALEEALSIQASPSSPPAAF     453
Rattus_norvegicus_(Rat)                                                                 KEQLTTEAKELRQKVKYLQDQLSPLMRQREYQEKEIQRLNKALEEALSIQASPSSPPAAF     455
Felis_catus_(Cat)_(Felis_silvestris_catus)                                              KEQLTAEAKELHQKVKYLQDQLSPLTRQREYQEKEIQRLNKALEEALSIQASPSSPPAAF     441
Pan_troglodytes_(Chimpanzee)                                                            KEQLTAEAKELRQKVKYLQDQLSPLTRQREYQEKEIQRLNKALEEALSIQTPPSSPPTAF     440
Gallus_gallus_(Chicken)                                                                 KDRLGMEVRDLQQRMRFLQEQLAPVTRQREYQEKEIQRLNKALEEALNVQASPPP---IF     417
Anolis_carolinensis_(Green_anole)_(American_chameleon)                                  KERLSAEAKDLKQRVLLQDQLTSITKQKEYQEKEILRLNKALEEALAFQASAPP-QSAF     422
Monodelphis_domestica_(Gray_short-tailed_opossum)                                       KERLTAEGKDLRQRAKYLQDQLTPLTRQREYQEKEIQRLNK------------------     422
Ictidomys_tridecemlineatus_(Thirteen-lined_ground_squirrel)_(Spermophilus_tridecemlineatus)   KEQLTAEAKELRQKVKYLQDQLSPLTRQREYQEKEIQRLNKALEEALSIQASPSSPPTAF     442
Myotis_lucifugus_(Little_brown_bat)                                                     KEQLSAEAKELRQKVRYLQDQLSPLTRQRDYQEKEIQRLNKALEEALSIQASPSSPPTAF     446
Otolemur_garnettii_(Small-eared_galago)_(Garnett's_greater_bushbaby)                    KEQLTAEAKELRQKVKYLQDQLSPLTRQREYQEKEIQRLNKALEEALSIQAPPSSPPTAF     441
Mustela_putorius_furo_(European_domestic_ferret)_(Mustela_furo)                         KEQLTAEAKELRQKVKHLQDQLSPLTRQREYQEKEIQRLNKALEEALSIQASPSSPPTAF     441
Ficedula_albicollis_(Collared_flycatcher)_(Muscicapa_albicollis)                        KERLAMEVRDLQQRMRFLQEQLAPVTRQREYQEKEIQRLNKALEEALNVQASPPP---IF     391
Chlorocebus_sabaeus_(Green_monkey)_(Cercopithecus_sabaeus)                              KEQLTAEAKELRQKVKYLQDQLSPLTRQREYQEKEIQRLNKALEEALSIQTPPSSPPTAF     441
Taeniopygia_guttata_(Zebra_finch)_(Poephila_guttata)                                    KERLAMEVRDLQQRMRFLQEQLAPVTRQREYQEKEIQRLNKALEEALNVQASPPP---IF     431
Pongo_abelii_(Sumatran_orangutan)_(Pongo_pygmaeus_abelii)                               KEQLTAEAKELRQKVKYLQDQLSPLTRQREYQEKEIQRLNKALEEALSIQSPPSSPPTAF     398
Macaca_fascicularis_(Crab-eating_macaque)_(Cynomolgus_monkey)_                          KEQLTAEAKELRQKVKYLQDQLSPLTRQREYQEKEIQRLNKALEEALSIQTPPSSPPTAF     441
Pelodiscus_sinensis_(Chinese_softshell_turtle)_(Trionyx_sinensis)                       KERLAGELQELKQRVRFLQEQLAPVTKQREYQERIQRLNKVGSLAAPAPDKP-----RA     421
Bos_taurus_(Bovine)                                                                     REHLTAEAKELRQKVKYLQDQLSPLTRQREYQEKEIQRLNKALEEALSIQASPSS-PTAF     438
Pan_paniscus_(Pygmy_chimpanzee)_(Bonobo)                                                KEQLTAEAKELRQKVKYLQDQLSPLTRQREYQEKEIQRLNKALEEALSIQTPPSSPPTAF     440
Capra_hircus_(Goat)                                                                     REHLTAEAKELRQKVKYLQDQLSPLTRQREYQEKEIQRLNKALEEALSIQASPSS-PTAF     423
Danio_rerio_(Zebrafish)_(Brachydanio_rerio)                                             KECLTSETCELKQKVRYLQDQLLPLTKQREYQEKEIQRLNRALEEALNLHSPSSTQPGMN     450
Xenopus_tropicalis_(Western_clawed_frog)                                                IQKLESEVRELKQKNKFLHEQLSSTSKQREYQEREISRLNKALEEALNMQGSGPCSPSLI     451
                                                                                         : *  *  :*:*: : *::**     :*::***:** ***:
```

```
Homo_sapiens_(Human)                                                                         GSPEGAGALLRKQELVTQNELLKQQVKIFEEDFQRERSDRERMNEEKEELKKQVEKLQAQ    500
Mus_musculus_(Mouse)                                                                         GSPEGVGGHLRKQELVTQNELLKQQVKIFEEDFQRERSDRERMNEEKEELKKQVEKLQAQ    513
Rattus_norvegicus_(Rat)                                                                      GSPEGVGGHLRKQELVTQNELLKQQVKIFEEDFQRERSDRERMNEEKEELKKQVEKLQAQ    515
Felis_catus_(Cat)_(Felis_silvestris_catus)                                                   GGPEGAGGLLRKQELVTQNELLKQQVKIFEEDFQRERSDRERMNEEKEELKKQVEKLQAQ    501
Pan_troglodytes_(Chimpanzee)                                                                 GSPEGAGALLRKQELVTQNELLKQQVKIFEEDFQRERSDRERMNEEKEELKKQVEKLQAQ    500
Gallus_gallus_(Chicken)                                                                      AGTLEPAGKVPPQELLTQNELLKQQVKIFEEDFQRERSDRERMNEEKEELKQQLEKLQKQ    477
Anolis_carolinensis_(Green_anole)_(American_chameleon)                                       NSSADPSVNIRQQEVMTQNDLLKQQVKIFEEDFQRERSDRERMNEEKEELKRQVEKLQKQ    482
Monodelphis_domestica_(Gray_short-tailed_opossum)                                            -------------------------VKIFEEDFQRERSDRERMNEEKEELKKQLEKLQAQ    457
Ictidomys_tridecemlineatus_(Thirteen-lined_ground_squirrel)_(Spermophilus_tridecemlineatus)  GSPEGAAGLLRKQELVTQNELLKQQVKIFEEDFQRERSDRERMNEEKEELKKQVEKLQAQ    502
Myotis_lucifugus_(Little_brown_bat)                                                          GNPEGAGGLLRKQELVTQNELLKQQVKIFEEDFQRERSDRERMNEEKEELKKQVEKLQAQ    506
Otolemur_garnettii_(Small-eared_galago)_(Garnett's_greater_bushbaby)                         GSPEGAGGLLRKQELVTQNELLKQQVKIFEEDFQRERSDRERMNEEKEELKKQVERLQAQ    501
Mustela_putorius_furo_(European_domestic_ferret)_(Mustela_furo)                              GGPEGAGGLLRKQELVTQNELLKQQVKIFEEDFQRERSDRERMNEEKEELKKQVERLQAQ    501
Ficedula_albicollis_(Collared_flycatcher)_(Muscicapa_albicollis)                             A--MEPAGKLPQQELLTQNELLKQQVKIFEEDFQRERSDRERMNEEKEELKQQLEKLQKQ    449
Chlorocebus_sabaeus_(Green_monkey)_(Cercopithecus_sabaeus)                                   GSPEGAGALLRKQELVTQNELLKQQVKIFEEDFQRERSDRERMNEEKEELKKQVEKLQAQ    501
Taeniopygia_guttata_(Zebra_finch)_(Poephila_guttata)                                         A--MEPAGKLPQQELLTQNELLKQQVKIFEEDFQRERSDRERMNEEKEELKQQLEKLQKQ    489
Pongo_abelii_(Sumatran_orangutan)_(Pongo_pygmaeus_abelii)                                    GSPEGAGALLRKQELVTQNELLKQQVKIFEEDFQRERSDRERMNEEKEELKKQVEKLQAQ    458
Macaca_fascicularis_(Crab-eating_macaque)_(Cynomolgus_monkey)_                               GSPEGAGALLRKQELVTQNELLKQQVKIFEEDFQRERSDRERMNEEKEELKKQVEKLQAQ    501
Pelodiscus_sinensis_(Chinese_softshell_turtle)_(Trionyx_sinensis)                            CSAKDGAGMVSQ-AAVRAGE----------------------------------------    440
Bos_taurus_(Bovine)                                                                          GSPEGAGGLLRKQELLTQNELLKQQVKIFEEDFQRERSDRERMNEEKEELKKQVEKLQAQ    498
Pan_paniscus_(Pygmy_chimpanzee)_(Bonobo)                                                     GSPEGAGALLRKQELVTQNELLKQQVKIFEEDFQRERSDRERMNEEKEELKKQVEKLQAQ    500
Capra_hircus_(Goat)                                                                          GSPEGAGGLLRKQELLTQNELLKQQVKIFEEDFQRERSDRERMNEEKEELKKQVEKLQAQ    483
Danio_rerio_(Zebrafish)_(Brachydanio_rerio)                                                  --LGEGVANLRQQELHTQIAVLKEQVKIFEEDFRKERSDRERMNEEKEDLRRQVERLQGQ    508
Xenopus_tropicalis_(Western_clawed_frog)                                                     ASLEDPR-LPQREELQMQNDILKQQVKIFEEDFQKERRDRERMNEEKEELKRQLELLQTQ    510


Homo_sapiens_(Human)                                                                         VTLSNAQLKAFKDEE--------KAREALRQQKR------------------------K    527
Mus_musculus_(Mouse)                                                                         VTLTNAQLKTLKEEE--------KAKEALKQQKR------------------------K    540
Rattus_norvegicus_(Rat)                                                                      VTLTTAQLRTLKEEE--------KAKEALKQQKR------------------------K    542
Felis_catus_(Cat)_(Felis_silvestris_catus)                                                   VTLSNAQLKAFKDEE--------KTKEALKQQKR------------------------K    528
Pan_troglodytes_(Chimpanzee)                                                                 VTLSNAQLKAFKDEE--------KAREALRQQKR------------------------K    527
Gallus_gallus_(Chicken)                                                                      LAVSNNQLRASKDDCQREKEEKEKLKKMLKQHKQ-------------------------    511
Anolis_carolinensis_(Green_anole)_(American_chameleon)                                       LMLTNNQLRTFKEDYQKEKEEKDKYKKMLKQHKQ-------------------------    516
Monodelphis_domestica_(Gray_short-tailed_opossum)                                            VTLSNAQLKAFKEEE--------KAKEALRQQKR------------------------K    484
Ictidomys_tridecemlineatus_(Thirteen-lined_ground_squirrel)_(Spermophilus_tridecemlineatus)  VTLSNAQIKSFKEFE--------KAKETLKQQKR------------------------K    529
Myotis_lucifugus_(Little_brown_bat)                                                          VTLSNAQLKAFKDEE--------KAKEALKQQKR------------------------K    533
Otolemur_garnettii_(Small-eared_galago)_(Garnett's_greater_bushbaby)                         VTLSNAQLKAFKDEE--------KAREALKQQKR------------------------K    528
Mustela_putorius_furo_(European_domestic_ferret)_(Mustela_furo)                              VTLSNAQLKAFKDEE--------KTKEALKQQKR------------------------K    528
Ficedula_albicollis_(Collared_flycatcher)_(Muscicapa_albicollis)                             LVLSNNQLRASKDDCQREKEEKEKLKKLLKQHKQ-------------------------    483
Chlorocebus_sabaeus_(Green_monkey)_(Cercopithecus_sabaeus)                                   VTLSNAQVKAFKDEE--------KAREANRQQKR------------------------K    528
Taeniopygia_guttata_(Zebra_finch)_(Poephila_guttata)                                         LVLSSNQVSST-----GEKEEKEKLKKLLKQHKQ-------------------------    518
Pongo_abelii_(Sumatran_orangutan)_(Pongo_pygmaeus_abelii)                                    VTLSNAQLKAFKDDE--------KAREALRQQKR------------------------K    485
Macaca_fascicularis_(Crab-eating_macaque)_(Cynomolgus_monkey)_                               VTLSNAQLKAFKDEE--------KAREANRQQKR------------------------K    528
Pelodiscus_sinensis_(Chinese_softshell_turtle)_(Trionyx_sinensis)                            --RAAGDLRVFKDDYQREKEEKEKLKKLLKQHKQVGRAVGRSSSGRWSEGCEPHAQVQRL    498
Bos_taurus_(Bovine)                                                                          VTLSHAQLKAFKDEE--------KAKDALRQQKR------------------------K    525
Pan_paniscus_(Pygmy_chimpanzee)_(Bonobo)                                                     VTLSNAQLKAFKDEE--------KAREALRQQKR------------------------K    527
Capra_hircus_(Goat)                                                                          VTLSHAQLKAFKDEE--------KAKDALRQQKR------------------------K    510
Danio_rerio_(Zebrafish)_(Brachydanio_rerio)                                                  MTNLTNQLHQAQNECQRERAERCKLERLQMQHKQEGQQERR------------------    549
Xenopus_tropicalis_(Western_clawed_frog)                                                     LGQVNTQLRSCQEDLRREMESSRD---AV-----------------------------R    537
                                                                                                  ::                .

Homo_sapiens_(Human)                                                                         AKASGERYHV-EPHPEH---LCGAYPYAYPPMPAMVPHHGFEDWSQIRYPPPPMAMEHPP    583
Mus_musculus_(Mouse)                                                                         AKASGERYHM-EPHPEH---VCGAYPYAYPPMPAMVPHHAYKDWSQIRYPPPPVPMEHPP    596
Rattus_norvegicus_(Rat)                                                                      AKASGERYHV-EPHPEH---LCGAYPYAYPPMPAMVPHHAYKDWSQIRYPPPPVPMEHPP    598
Felis_catus_(Cat)_(Felis_silvestris_catus)                                                   AKASADRYHM-EPHPEH---LCGAYPYAYAPMPAMVPHHGFEDWSQIRYPPPPMAMEHPP    584
Pan_troglodytes_(Chimpanzee)                                                                 AKASGERYHV-EPHPEH---LCGAYPYAYPPMPAMVPHHGFEDWSQIRYPPPPMAMEHPP    583
Gallus_gallus_(Chicken)                                                                      --ASGERLHP-DPVPGPLGPACPMYQYQYSPPVPHPMYHGYDEWQQIRYPPAL-QGEHAP    567
Anolis_carolinensis_(Green_anole)_(American_chameleon)                                       --HSGDRLHQENPQPAPVAPVCPMYQFQYSPPAQAHLYRGYEDWQQIRYPPSALASEQPP    574
Monodelphis_domestica_(Gray_short-tailed_opossum)                                            MKASGERYHM-DPLPEH---ICGAYPFACQPVTPLLHHHGFEDWSEIRYPPPPMATEHQP    540
Ictidomys_tridecemlineatus_(Thirteen-lined_ground_squirrel)_(Spermophilus_tridecemlineatus)  AKASEERYHV-DPLPEH---LCGPYLYAYPPTPAMVPHHGFEDWSQIRYPPPPMAMEHPP    585
Myotis_lucifugus_(Little_brown_bat)                                                          AKASAERYHV-EPHPYF---G-----Y----YGPPVPHHGYEEWSQIRYPPPPMAMEHPP    580
Otolemur_garnettii_(Small-eared_galago)_(Garnett's_greater_bushbaby)                         AKASGERYHV-EPHPEH---LCGAYPYAYPPMPAMVPHHGFEDWSQIRYPPPPMAMEHPA    584
Mustela_putorius_furo_(European_domestic_ferret)_(Mustela_furo)                              AKASAEHYRM-EPHPEH---LCGAYPYAYAPMPAMVPHHGYEDWSQIRYPPPPMAMEHQP    584
Ficedula_albicollis_(Collared_flycatcher)_(Muscicapa_albicollis)                             --ASGERLHP-EPGPGPLGPACPMYQYQYSPPMAHPMYHGYDEWQQIRYPPAV-PGEHTQ    539
Chlorocebus_sabaeus_(Green_monkey)_(Cercopithecus_sabaeus)                                   AKASGERYHV-EPHPEH---LCGAYPYAYPPMPAMVPHHGFEDWSQIRYPPPPMAMEHPP    584
Taeniopygia_guttata_(Zebra_finch)_(Poephila_guttata)                                         --ASGERLHA-EPGPGPLGPACPMYQYQYSPPVAHPVYHGYEEWQQIRYPP---------    566
Pongo_abelii_(Sumatran_orangutan)_(Pongo_pygmaeus_abelii)                                    AKASGERYHV-EPHPEH---LCGAYPYAYPPMPAMVPHHGFEDWSQIRYPPPPMAMEHPP    541
Macaca_fascicularis_(Crab-eating_macaque)_(Cynomolgus_monkey)_                               AKASGERYHV-EPHPEH---LCGAYPYAYPPMPAMVPHHGFEDWSQIRYPPPPMAMEHPP    584
Pelodiscus_sinensis_(Chinese_softshell_turtle)_(Trionyx_sinensis)                            GGVSGERLHP-EPLQGPLGPACPVYQFQYSPPGPHHVYRGYEDWQQIRYPPSAMPGEHPP    557
Bos_taurus_(Bovine)                                                                          AKTSADRYHL-EPHPEH---LCGACPYAYPPMPPIGPPPGFEDWSQIRYPPPPTAMEHPP    581
Pan_paniscus_(Pygmy_chimpanzee)_(Bonobo)                                                     AKASGERYHV-EPHPEH---LCGAYPYAYPPMPAMVPHHGFEDWSQIRYPPPPMAMEHPP    583
Capra_hircus_(Goat)                                                                          AKTSADRYHL-EPHPEH---LCGACPYAYPPMPPIGPHPGFEDWSQIRYPPPPTAMEHPP    566
Danio_rerio_(Zebrafish)_(Brachydanio_rerio)                                                  --------TSDPTSNAPNGPMSPPYCG----PFVQVGHQGLEGW-PIHFPPRMPNISTAP    596
Xenopus_tropicalis_(Western_clawed_frog)                                                     KQALGERYIV-DPHMGH---VCPPYQYPYSPPG--LVYPAFEDW-QIRYPPPVVHPEHSQ    590
                                                                                                                                    . . *  *::**

Homo_sapiens_(Human)                                                                         PLPNSRLFHLP-EYTWRL-P-----CGGVRNPNQSSQVMDP--PTARPTEPESPKNDREG    634
Mus_musculus_(Mouse)                                                                         PHPNSRLFHL-PEYTWRP-PCAGIR-------NQSSQVMDP--PPDRPAEPESADNDCDG    645
Rattus_norvegicus_(Rat)                                                                      PHPNSRLFHL-PEYTWRP-PCAGIR-------NQNSQVMDP--PPDRPAEPESADNDCDG    647
Felis_catus_(Cat)_(Felis_silvestris_catus)                                                   PLPNSRLFHLP-EYTWRP-PC-----GGMR--NQSSQVMDS--PTARPAEPESTKNDREG    633
Pan_troglodytes_(Chimpanzee)                                                                 PLPNSLCPSLAPGHTQRL-PQTPSREEGVRNPNQSSQVMDP--PTARPTEPGEPLILSLG    640
Gallus_gallus_(Chicken)                                                                      GQ-NFHHYP-PPEYPWRP-PCAMTRS-------QNATAVPG--VKPVPKDLDQA--GPAL    613
Anolis_carolinensis_(Green_anole)_(American_chameleon)                                       VQ-NYHRFP-PPEYNWYPPPCVLSQN-------QNSQAVDG--VKQFSKDSDPT--GLGL    621
Monodelphis_domestica_(Gray_short-tailed_opossum)                                            PIPNSRLFHL-PEYAWRS-PCGVMRNQSSQVDAA---LVQP--SDPEPTDHGLPKN----    589
Ictidomys_tridecemlineatus_(Thirteen-lined_ground_squirrel)_(Spermophilus_tridecemlineatus)  PLPNSRLFHL-PEYTWRP-PCGTMR-------NQSAQVTDP--PSTRPTDPESAKNDHEG    634
Myotis_lucifugus_(Little_brown_bat)                                                          PLPNSHLYPM-PGYTWRP-PY-----GGMR--NQTSQVMDS--PTARPAEPDSAKNDRKG    629
Otolemur_garnettii_(Small-eared_galago)_(Garnett's_greater_bushbaby)                         PLPNSRLFHLP-EYTWRP-PY-----GGIR--NQSSQVMDP--SPARPVEPESTKNDREG    633
Mustela_putorius_furo_(European_domestic_ferret)_(Mustela_furo)                              PLPNSRLFHLP-EYTWRP-PC-----GGMR--NQSSQVMDP--PPARPVEPESTKNDREG    633
Ficedula_albicollis_(Collared_flycatcher)_(Muscicapa_albicollis)                             GQ-NFHHFP-PPEYPWRP-PCAMAHS-------QNAQPVAG--LKPVPKDLEQA--GPGL    585
 Chlorocebus_sabaeus_(Green_monkey)_(Cercopithecus_sabaeus)                                   PLPNSRLFHLP-EYTWRL-P-----CGGVRNPNQSSQMMDS--PTARPTEPESPKNDREG    635
Taeniopygia_guttata_(Zebra_finch)_(Poephila_guttata)                                         ------------------------------------------------------------    566
Pongo_abelii_(Sumatran_orangutan)_(Pongo_pygmaeus_abelii)                                    PLPNSRLFHLP-EYTWRL-P-----CGGIRNPNQSSQVMDP--PTARPTEP---------    583
Macaca_fascicularis_(Crab-eating_macaque)_(Cynomolgus_monkey)_                               PLPNSRLFHLP-EYTWRL-P-----CGGVRNPNQSSQMMDS--PTARPTEPESPKNDREG    635
Pelodiscus_sinensis_(Chinese_softshell_turtle)_(Trionyx_sinensis)                            VQ-NFHHFP-PPEYAWRP-PCAVSRT-------QNSQAVEG--VKPLPKEPAEEQGGREV    605
Bos_taurus_(Bovine)                                                                          PLPNSRLFRL-NTPGGC--PVEGY-------------VIRA--PK---------------    608
Pan_paniscus_(Pygmy_chimpanzee)_(Bonobo)                                                     PLPNSLCPSLAPGHTQRL-PQTPSREEGVRNPNQSSQVMDP--PTARPTEPGEPLILSLG    640
Capra_hircus_(Goat)                                                                          PLPNSRLFRL-PEYTWRM-PFGGVR-------NQSSQVMDL--PTARPTEPESTKNDREG    615
Danio_rerio_(Zebrafish)_(Brachydanio_rerio)                                                  ---GRDFQPVNPGFPWQS-SFPQPRG--SRGQAD--TAR----APPETAEMGATGFGKRE    644
Xenopus_tropicalis_(Western_clawed_frog)                                                     IQ-DLNNVP-PPAYPWRMATIL-PRMQNSKNKKK-EQDIAGSGTQNAPRQT---------    637
```

```
Homo_sapiens_(Human)                                                                             PQ-------      636
Mus_musculus_(Mouse)                                                                             PQ-------      647
Rattus_norvegicus_(Rat)                                                                          PQ-------      649
Felis_catus_(Cat)_(Felis_silvestris_catus)                                                       PQ-------      635
Pan_troglodytes_(Chimpanzee)                                                                     PPWGR----      645
Gallus_gallus_(Chicken)                                                                          P--------      614
Anolis_carolinensis_(Green_anole)_(American_chameleon)                                           PKNQRPT--      628
Monodelphis_domestica_(Gray_short-tailed_opossum)                                                ---------      589
Ictidomys_tridecemlineatus_(Thirteen-lined_ground_squirrel)_(Spermophilus_tridecemlineatus)      PQ-------      636
Myotis_lucifugus_(Little_brown_bat)                                                              PQ-------      631
Otolemur_garnettii_(Small-eared_galago)_(Garnett's_greater_bushbaby)                             PQ-------      635
Mustela_putorius_furo_(European_domestic_ferret)_(Mustela_furo)                                  PQ-------      635
Ficedula_albicollis_(Collared_flycatcher)_(Muscicapa_albicollis)                                 P--------      586
Chlorocebus_sabaeus_(Green_monkey)_(Cercopithecus_sabaeus)                                       PQ-------      637
Taeniopygia_guttata_(Zebra_finch)_(Poephila_guttata)                                             ---------      566
Pongo_abelii_(Sumatran_orangutan)_(Pongo_pygmaeus_abelii)                                        ---------      583
Macaca_fascicularis_(Crab-eating_macaque)_(Cynomolgus_monkey)_                                   PQ-------      637
Pelodiscus_sinensis_(Chinese_softshell_turtle)_(Trionyx_sinensis)                                PV-------      607
Bos_taurus_(Bovine)                                                                              ---------      608
Pan_paniscus_(Pygmy_chimpanzee)_(Bonobo)                                                         PPWGR----      645
Capra_hircus_(Goat)                                                                              PQ-------      617
Danio_rerio_(Zebrafish)_(Brachydanio_rerio)                                                      RQNIDPGKH      653
Xenopus_tropicalis_(Western_clawed_frog)                                                         ---------      637
```

Figure S1. Clustal Omega alignment of TNIP1. Multiple sequence alignments of TNIP1 homologs. * (asterisk) denotes positions featuring a single, fully conserved residue, : (colon) denotes conservation of residues featuring extremely similar properties, . (period) denotes conservation of residues featuring weakly similar properties.

Common Name	Species name	Uniprot identifier
Bonobo	*Pan paniscus*	A0A2R8ZVM7
Bovine	*Bos taurus*	A0A3S5ZPJ2
Brown bat	*Myotis lucifugus*	G1PDF5
Bushbaby	*Otolemur garnettii*	H0WG84
Cat	*Felis silvestris catus*	M3WHS7
Chimpanzee	*Pan_troglodytes*	A0A2I3SHK0
Chicken	*Gallus gallus*	F1NQ66
Chinese softshell turtle	*Pelodiscus sinesis*	K7FF90
Collared flycatcher	*Ficedula albicollis*	U3K078
Ferret	*Mustela putorius furo*	M3YQJ3
Goat	*Capra hircus*	A0A452G1D6
Green anole	*Anolis carolinensis*	G1KKH7
Green monkey	*Chlorocebus sabaeus*	A0A0D9RFW6
Human	*Homo Sapiens*	Q15025
Mouse	*Mus musculus*	Q9WUU8
Opossum	*Monodelphis domestica*	F7GJZ2
Rat	*Rattus norvegicus*	D3ZHV1
Squirrel	*Ctidomys tridecemlineatus*	I3M719
Sumatran orangutan	*Pongo abelii*	H2PH42
Western clawed frog	*Xenopus tropicalis*	Q08CX3
Zebra finch	*Taeniopygia guttata*	H0YRE9
Zebrafish	*Danio rerio*	A1L1N2

Table S1. Species listed which were used for *in silico* assessments in Figure 1, Supplemental table S2 and Supplemental figure S2.

A

Percent Identity (%)	Human	Green Monkey	Crab-eating Macaque	Chimpanzee	Orangutan	Bonobo
Human	100	97.48	97.48	96.70	98.80	96.86
Green Monkey	97.48	100	98.90	94.81	97.43	94.65
Crab-eating Macaque	97.48	98.90	100	94.81	97.94	94.65
Chimpanzee	96.70	94.81	92.11	100	96.91	99.84
Orangutan	98.80	97.43	97.94	96.91	100	97.08
Bonobo	96.86	94.65	94.65	99.84	97.08	100

B

Percent Identity (%)	Human	Zebrafish	Collared Flycatcher	Zebra Finch	Western Clawed Frog
Human	100	46.48	56.99	54.07	50.59
Zebrafish	46.48	100	47.03	44.76	40.47
Collared Flycatcher	56.99	47.03	100	85.33	49.94
Zebra Finch	54.07	44.76	85.33	100	47.50
Western Clawed Frog	50.59	40.47	49.91	47.50	100

Table S2. Percent identity matrix derived from alignment of TNIP1 in 23 species. (a) Tables showing calculated similarity in amino acid identity with the 5 highest scorers and **(b)** 4 lowest scorers (less than 60% identity match) as compared to human. Percent identity scores were calculated using standard settings within the Clustal Omega sequence alignment algorithm.

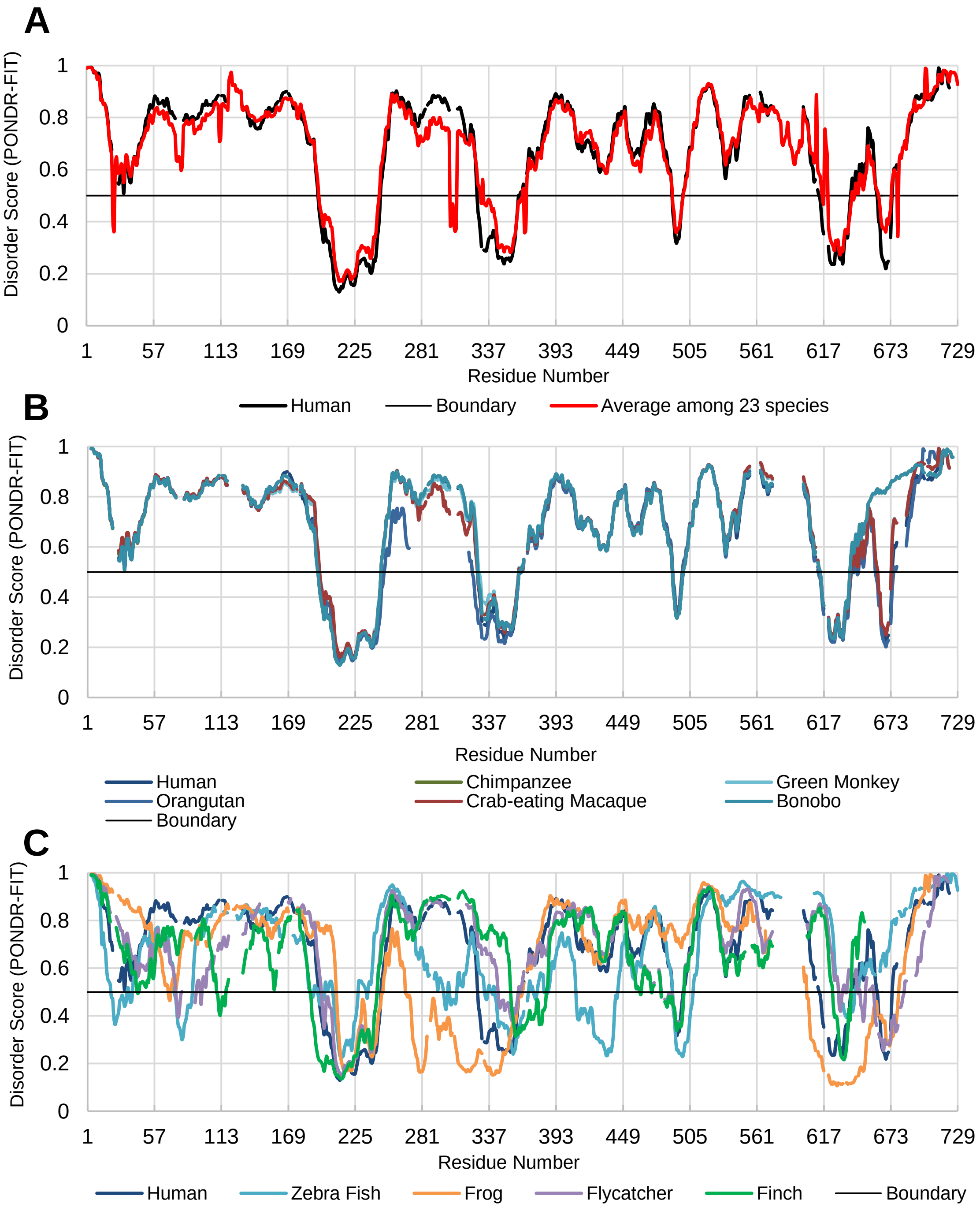

Figure S2. Species comparison of TNIP1 homologs by percent identity similarity. (a) PONDR-FIT scores for human TNIP1 versus average PONDR-FIT of all 23 species used in the Clustal Omega alignment. **(b)** Top five and **(c)** bottom four species in amino acid sequence homology, compared with human, as determined by the percent identity similarity post alignment with Clustal Omega (sequence identity less than 60%). Boundary line (0.5) delineates prediction of disorder (any residue score above 0.5 predicts disorder in region). Gaps were introduced based on alignment data generated using Clustal Omega with default parameters.

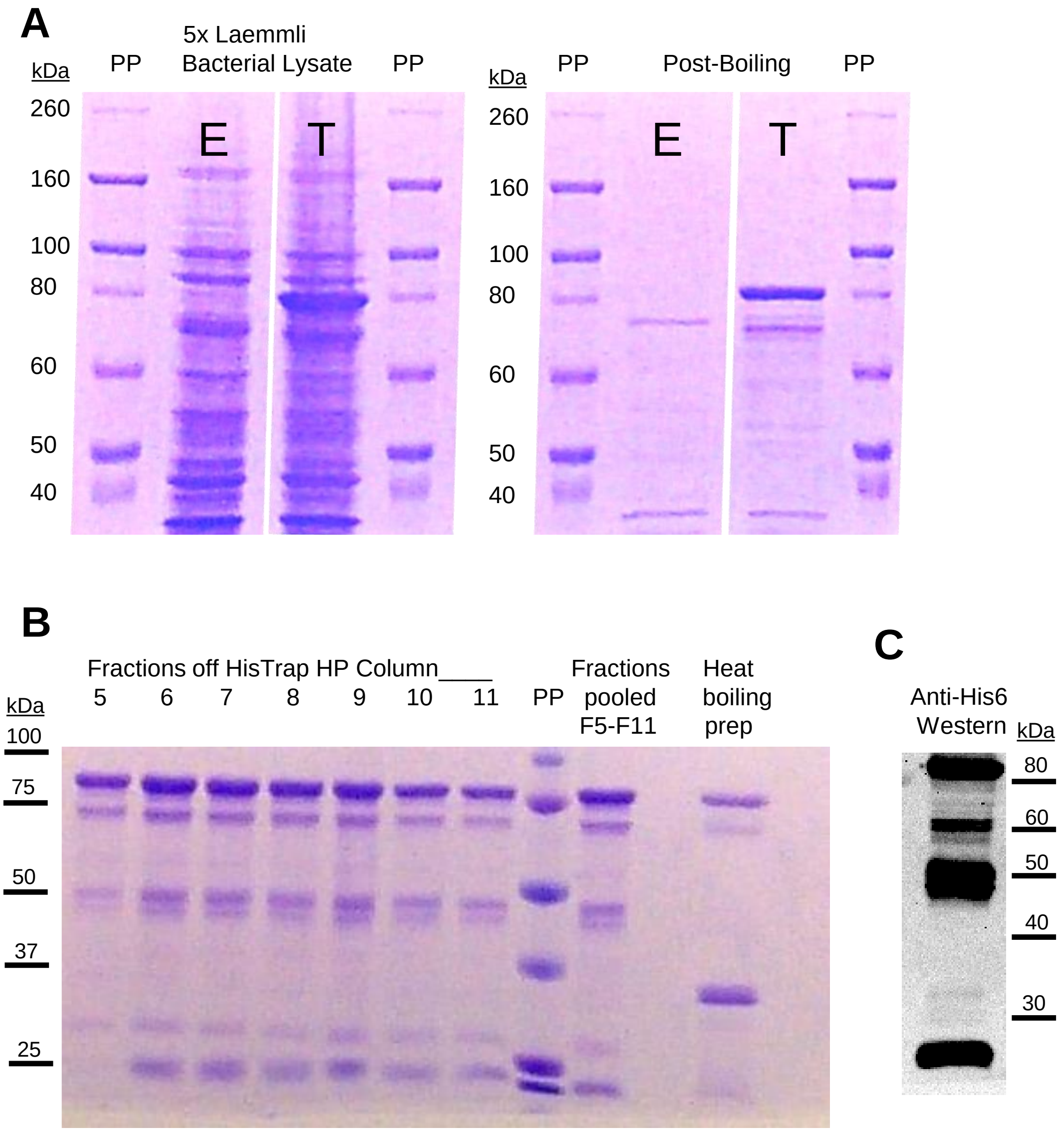

Figure S3. Expression and purification of full-length TNIP1. pET28a empty vector (E) and pET28a – TNIP1 (T) were induced with 0.1 mM IPTG and expression was allowed to go for 4 hours at 30 °C before bacterial pellets were collected. Lysis occurred with direct addition of **(a)** 5X Laemmli buffer or resuspension at 1/20 (w/v) in 20 mM sodium phosphate buffer (pH 8.0), 500 mM NaCl, 20 mM imidazole before heating in 99 °C water bath for 20 minutes followed by immediate cooling on ice for 10 minutes. **(b)** Pellets were clarified at 28,000x *g* for 35 minutes before injection onto pre-calibrated 5 mL HisTrap HP column at 1 mL/minute and eluted with 20 mM sodium phosphate buffer (pH 8.0), 500 mM NaCl, 300 mM imidazole, and 10% glycerol over seven fractions (sample post clarification immediately prior to injection onto column loaded in last lane labeled as "heat boiling prep"). **(c)** Identification of pooled elution from panel b was performed using western blot analysis with anti-His6 antibody. PP – pre-stained SDS-PAGE protein standards.

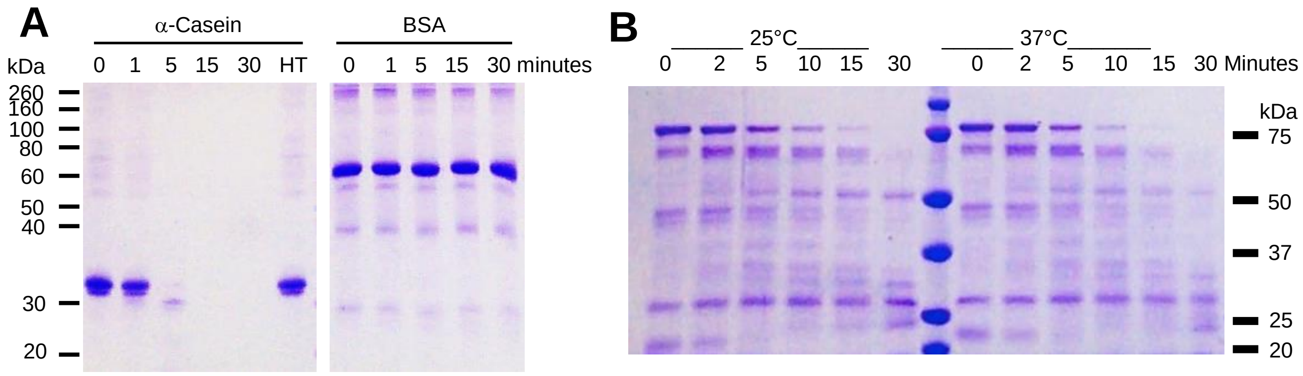

Figure S4. Limited proteolysis of full-length TNIP1 and control proteins. (a) Limited proteolysis of positive and negative controls α-casein and BSA, respectively. Proteolysis was performed with 500:1 w/w ratio of protein substrate to trypsin. Experiments were performed at 37°C. **(b)** Limited proteolysis performed with full-length TNIP1 with 250:1 TNIP1 to chymotrypsin ratio (w/w) at 25 °C and 37 °C. Samples collected over 30 minutes with reactions quenched with addition of 5X Laemmli buffer and heating at 95 °C for 5 minutes prior to loading 6 μg on a acrylamide gel for analysis by SDS-PAGE. HT – heat control with no enzyme present.

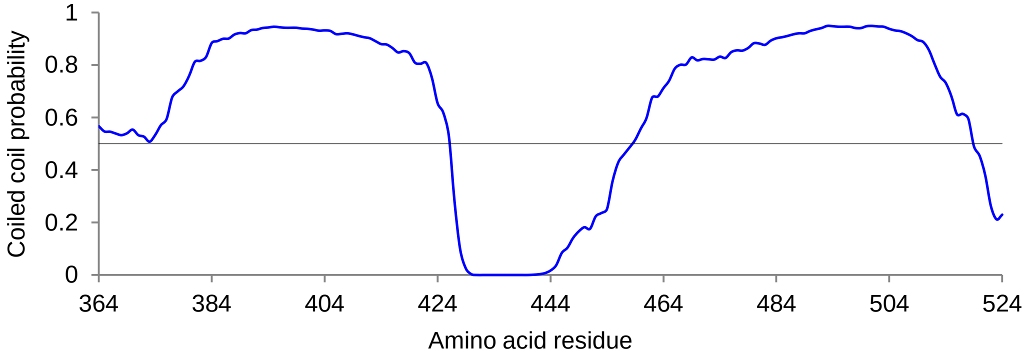

Figure S5. *In silico* prediction of coiled-coil domain across A20 and ubiquitin binding domains of TNIP1. DeepCoil prediction scores represented as coiled-coil probability across AA364-524 of TNIP1.

ENSP00000430760, ENSP00000428187, ENSP00000317891

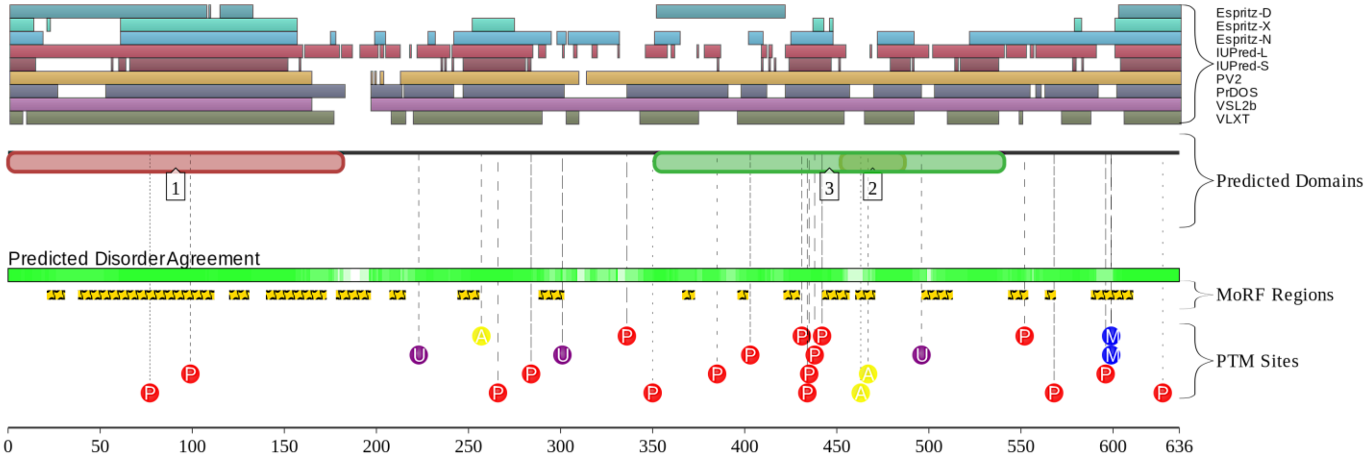

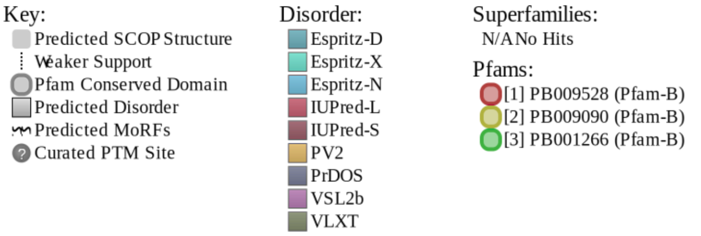

Position	AA	Context
431	T	LSIQ**T**PPSS
434	S	QTPP**S**SPPT
435	S	TPPS**S**PPTA
438	T	SSPP**T**AFGS
442	S	TAFG**S**PEGA

Figure S6. D2P2 analysis of human TNIP1 amino acid sequence. The UniProt sequence Q15025 for human TNIP1 was submitted to D2P2 (http://d2p2.pro/). Aligned results are presented for the nine disorder predictors it examines with extent of disorder agreement among them indicated by the intensity of green in the Predicted Disorder Agreement bar representing the entire length of the protein. Predicted molecular recognition features (MoRF) regions and post-translational modification (PTM) sites are also reported. The inset table summarizes predicted phosphorylation sites within the AHD1-UBAN region of TNIP1 AA417-509.

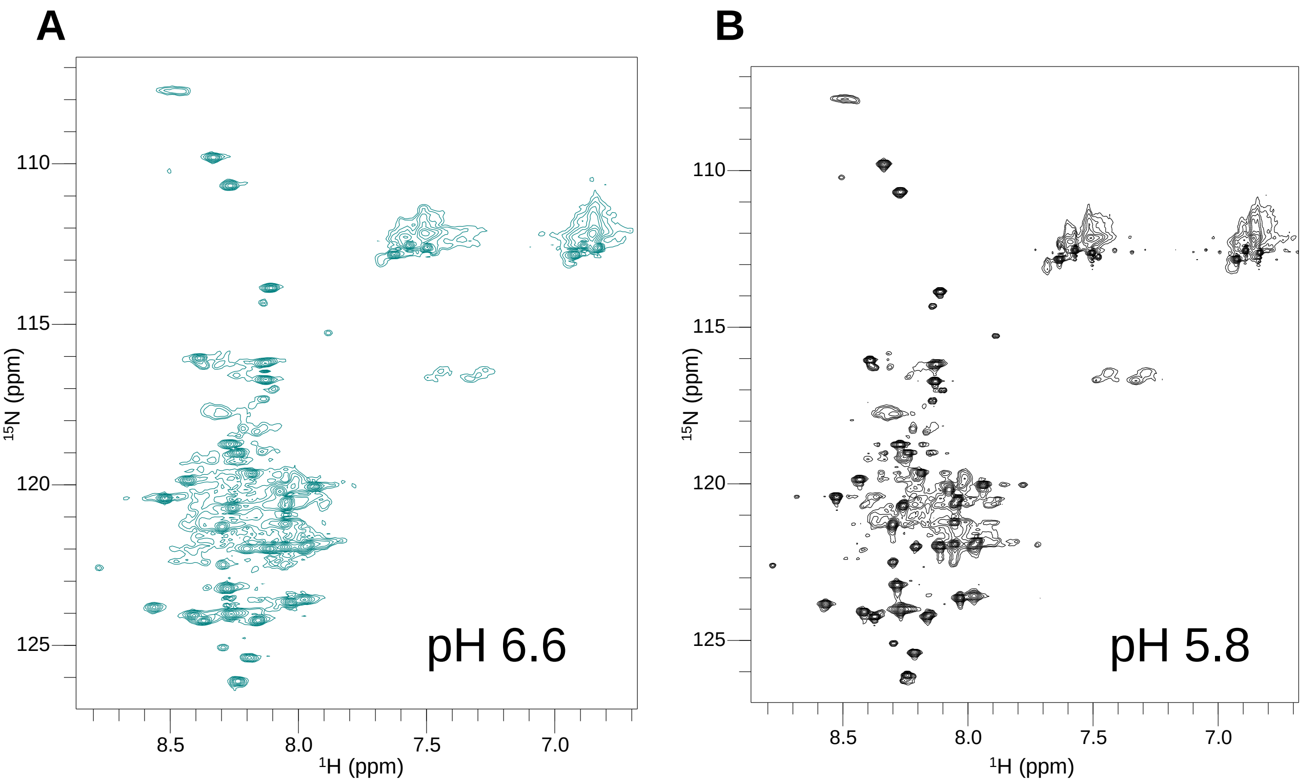

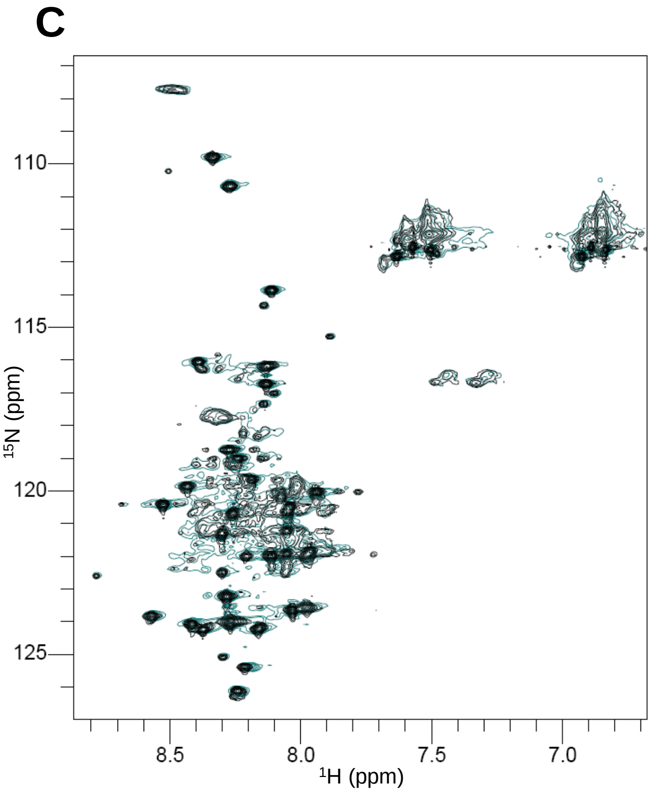

Figure S7. ^{1}H – ^{15}N HSQC spectra of AHD1-UBAN. Spectra of TNIP1$^{417-509}$ collected at **(a)** pH 6.6 and **(b)** pH 5.8 in 20 mM sodium phosphate buffer and 50 mM NaCl. **(c)** Overlay of spectra from panel (a) and (b).
